# Niban apoptosis regulator 1 promotes gemcitabine resistance by activating the focal adhesion kinase signaling pathway in bladder cancer

**DOI:** 10.7150/jca.66248

**Published:** 2022-01-09

**Authors:** Shiyu Tong, Hongling Yin, Jun Fu, Yangle Li

**Affiliations:** 1 Department of Urology, Xiangya Hospital, Central South University, Changsha, China.; 2 Department of Pathology, Xiangya Hospital, Central South University, Changsha, China.; 3 Department of Neurosurgery, Sanbo Brain Hospital, Beijing, China.

**Keywords:** bladder cancer, NIBAN1, FAK, gemcitabine, chemoresistance

## Abstract

Although intravesical gemcitabine (GEM) chemotherapy (IGC) can effectively reduce the recurrence risk of non-muscle invasive bladder cancer (NMIBC), the development of GEM resistance may occur and result in cancer recurrence and disease progression. Herein, a label-free proteomics approach was used to characterize the proteomic profiles of primary/post-IGC recurrent NMIBC. A total of 218 proteins were found to be differentially expressed in paired primary and post-IGC recurrent NMIBC. Kyoto Encyclopedia of Genes and Genomes pathway analysis revealed that multiple signaling pathways including “focal adhesion” were highly enriched in recurrent NMIBC. Niban apoptosis regulator 1 (NIBAN1) was identified as the top upregulated protein in recurrent NMIBC. Highly increased NIBAN1 expression was observed in a number of GEM-resistant cancer cell lines and in post-IGC recurrent NMIBC specimens. Manipulation of NIBAN1 expression affected the chemosensitivity to GEM in bladder cancer cell models. Moreover, NIBAN1 also regulated focal adhesion/focal adhesion kinase (FAK) signaling activation in bladder cancer cell lines. Highly elevated FAK (pY397) expression was observed in post-IGC recurrent NMIBC specimens, which was positively correlated with NIBAN1 expression. Knockdown of FAK markedly attenuated GEM resistance in GEM-resistant bladder cancer cells. *In vivo* studies demonstrated that knockdown of NIBAN1 disrupted FAK signaling and sensitized GEM-resistant bladder cancer cells to GEM treatment. Our findings suggest that NIBAN1 might regulate FAK signaling activation to promote GEM resistance in bladder cancer. Targeting NIBAN1/FAK signaling may help sensitize bladder cancer cells to GEM treatment.

## Introduction

Bladder cancer is the seventh most common type of cancer (7.68/100,000), and is the ninth leading cause of cancer-related deaths in China 1. Non-muscle invasive bladder cancer (NMIBC) comprises more than 70% of all newly diagnosed cases 2. The high rate of disease recurrence (40-80%) is a major challenge for NMIBC treatment 2. Intravesical gemcitabine (GEM) chemotherapy (IGC) has been investigated as an emerging adjuvant treatment for NMIBC. Compared with traditional Bacille Calmette-Guerin immunotherapy, IGC is a promising therapy with favorable efficacy in reducing the risk of recurrence and disease progression 3. However, about 20-50% NMIBC recurs within 2 years after IGC, which might be caused by intrinsic/acquired resistance to GEM 4, 5. Although *in vitro* studies have identified a number of genes (e.g., ribonucleotide reductase catalytic subunit M1, methionine adenosyltransferase 1A, DNA damage regulated autophagy modulator 2) involved in the regulation of GEM resistance in bladder cancer 6-8, the molecular mechanisms of GEM resistance remain largely unknown. The comprehensive characterization of expression profiles associated with GEM resistance in clinically relevant samples might help with the identification of novel regulators and design of therapeutic strategies to reduce the risk of recurrence in NMIBC.

Label-free quantitative proteomics approaches have been widely used in various applications including identifying expression profiles in different biological processes, searching for cancer biomarkers, and studying protein interaction networks 9. These approaches are rapid and sensitive to analyze various clinical sample types including fresh/cryopreserved tissues, serum, and archival formalin-fixed, paraffin-embedded (FFPE) tissues 10-12. Recently, label-free quantitative proteomics approaches have been used to discover drug resistance-related mechanisms in many cancers. Chu *et al.* 13 identified folate receptor 1 as a driver of sorafenib resistance in hepatocellular carcinoma cells based on label-free quantitative proteomics analysis. In multiple myeloma, exportin 1 was identified as a critical player for bortezomib resistance through label-free proteomics approaches 13. However, there is still a lack of proteomics studies on GEM resistance in NMIBC.

In this study, we characterized the proteomic profiles of primary and post-IGC recurrent NMIBCs using a label-free quantitative proteomics approach. Niban apoptosis regulator 1 (NIBAN1) was identified as the top upregulated protein in post-IGC recurrent NMIBC. Although NIBAN1 is involved in the carcinogenesis of renal and thyroid cancer 14, 15, its role in cancer drug resistance has not yet been reported. Our present findings suggest that NIBAN1 might be a novel regulator of GEM resistance in bladder cancer, which may help develop novel therapeutic strategies in the future.

## Materials and Methods

### Clinical specimens

This retrospective study was approved by the research ethics committee of Xiangya Hospital, Central South University (Changsha, China). Archival FFPE bladder cancer specimens were obtained from patients with following criteria: inclusion criteria: patients were initially diagnosed with NMIBC (Ta-T1) at Xiangya Hospital, Central South University (2017-2020); these patients had no prior history of treatment before receiving TURBT. Exclusion criteria: Patients with any prior nonurothelial or muscle-invasive bladder cancer; patients with previous or concurrent upper urinary tract or prostatic urethral urothelial cancer, previous pelvic radiotherapy for any malignancy, or prior treatment for any malignancy within 5 years.

All eligible patients had undergone initial TURBT and confirmed cancer free with subsequent second cystoscopy and biopsy before commencing intravesical gemcitabine chemotherapy (IGC). These patients received IGC consisting of eight weekly intravesical instillations (induction course). Gemcitabine was instilled at a dose of 2,000 mg per treatment with 1 h of retention time. Patients received monthly maintenance treatment (8-10 courses) depending on their recurrence risk profile, provided there was no evidence of recurrence on subsequent cystoscopies. The clinical follow-up was prescribed, consisting of urine cytology and cystoscopy per 3 months, and upper tract computed tomography urography during the first year, every 6 months for the next 2 years, and then yearly thereafter 16. The presence of disease recurrence was defined as histology proven tumor recurrence (any grade) or appearance of carcinoma in situ based on follow-up cystoscopy and pathological interpretation 16. The archival FFPE specimens of these paired primary/recurrent tumors were collected for label-free proteomics studies. The clinicopathological information associated with these primary/recurrent specimens is provided in **Table S1**.

### Reagents and Cell lines

Primary antibodies were provided by the following sources: NIBAN1 and β-Actin (Proteintech, Rosemont, IL, USA); p-FAK (Y397) and FAK (Affinity Biosciences); p-SRC (Y416), SRC, p-AKT (S473), and AKT (Cell signaling, Danvers, MA). Gemcitabine (GEM) was obtained from Selleckchem (Houston, TX, USA). Human bladder cancer cell lines T24 and 5637 were purchased from the Type Culture Collection of the Chinese Academy of Sciences, Shanghai, China. GEM-resistant T24 subline T24GR was cultured as described by Xie et al 17. Lentiviral expression Lv105 plasmids encoding empty vector (EV) or NIBAN1 were obtained from Genecopoeia Inc. Lentiviral shRNA plasmids encoding scramble control, shNIBAN1, and shFAK/PTK2 were also provided by Genecopoeia Inc. The oligo sequences and the methods for lentivirus packaging and infection could be found in '**Supplementary methods**'.

### Protein extraction, trypsin digestion and label-free proteomic analysis

**Figure 1:**
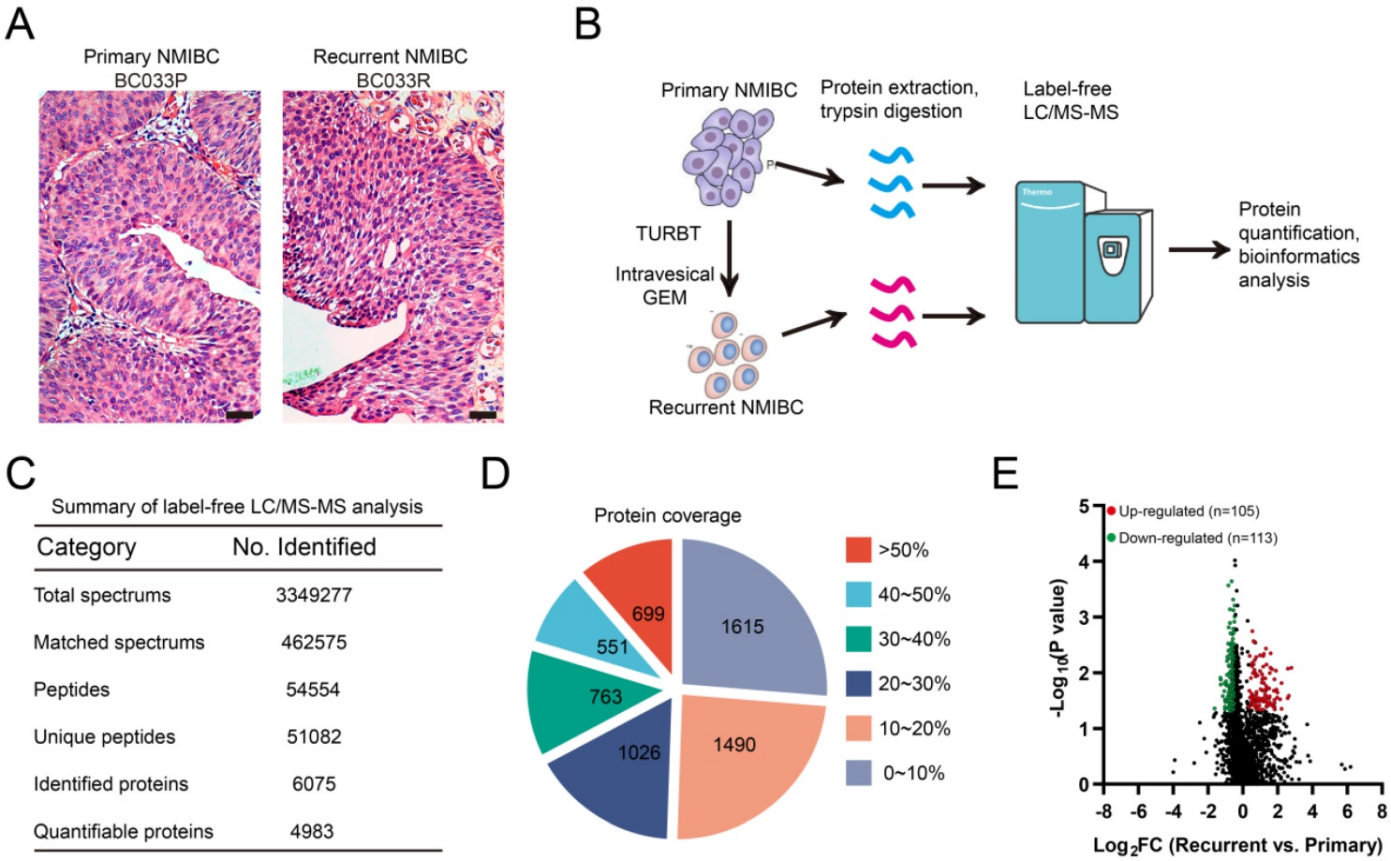
** Label-free proteomics analysis identifies recurrence-related proteomic profiles in NMIBC. A**. Representative micrographs of hematoxylin and eosin staining of paired primary and post-IGC recurrent NMIBCs. Bars: 100 μm. **B**. The workflow for label-free proteomics analysis on eight paired primary/post-IGC recurrent NMIBCs. **C**. Summary of label-free quantitative proteomics analysis. **D.** Protein coverage of identified proteins. **E**. Volcano plot of differentially expressed protein in recurrent versus primary comparison.

The experimental procedure was shown in **Fig. 1B**. FFPE samples were deparaffinized, lysed and de-crosslinked, followed by centrifugation at 12,000 g for 10 min. After trypsin digestion, the tryptic peptides were desalted with SPE column and dried by vacuum centrifuging. The tryptic peptides were dissolved in 0.1% formic acid, and then loaded onto a nanoElute UHPLC system, followed by the timsTOF Pro mass spectrometry in parallel accumulation serial fragmentation (PASEF) mode. The resulting MS/MS data were processed using MaxQuant search engine (v.1.6.15.0). Tandem mass spectra were searched against the human database (SwissProt, 20395 entries). More experimental details regarding protein extraction, trypsin digestion, and LC-MS/MS Analysis could be found in '**Supplementary methods**'.

### Cell viability analysis

Bladder cancer cells were plated in 96-well culture plates in triplicate (2×10^3^ cells/well). Cell viability was evaluated with a CCK-8 assay kit at 48 hours after GEM treatment. Phosphate buffered saline (PBS) was used as a treatment control. The CCK-8 absorbance was measured by a Multiskan MK3 microplate reader (OD_450_). The 50% inhibiting concentration (IC_50_) values were calculated in GraphPad prism 8 software.

### Soft agar assay

Soft agar assay was used to examine the *in vitro* clonogenesis of bladder cancer cells after GEM treatment 18. Briefly, the 1.5 mL culture medium with 0.5% agar was first plated into each well of a 6-well culture plate. After the agar solidified, each well received another 1.5 mL of 0.35% agar in culture medium containing 5×10^3^ cells with or without GEM. PBS was used as a treatment control. After 10~12 days, colonies in each group were counted.

### Western blotting

Western blot was conducted as by Fu et al 18. Protein lysates (25 μg) was separated by 10% SDS-PAGE and transferred to Hybond-P PVDF membranes (Millipore). Blots were detected by antigen-antibody reaction, and were visualized with an enhanced chemiluminescence detection reagent. β-Actin was used as a loading control.

### Caspase-3 activity assay

Apoptosis of cells was measured using an Abcam caspase-3 colorimetric assay kit as described previously 18. The Caspase-3 activity was evaluated at 48 hours after GEM treatment in bladder cancer cells. PBS was used as a treatment control. The optical density (OD_405_) of enzyme reactions was measured by a Multiskan MK3 microplate reader.

### Immunohistochemistry (IHC)

Chemo-naïve primary (n=30) and post-IGC recurrent (n=25) NMIBC specimens were used for IHC analysis. IHC procedure was conducted as described previously 18. Antigen-antibody reactions (Antibody dilution for NIBAN1: 1:100; dilution for p-FAK: 1:200) were visualized by exposure to chromogen substrate. The rabbit isotype IgG was used as a negative control. The scoring criteria for IHC results are described by Tan et al 19.

### RNA sequencing

The mRNA was extracted from T24GR cells with or without NIBAN1 knockdown. The mRNA libraries were then constructed and RNA sequencing was performed on an Illumina NovaSeq6000 platform (HaploX, Shenzhen, China). The expression level of protein-coding genes was calculated as Fragments Per Kilobase of exon model per Million mapped fragments (FPKM) value.

### Gene Expression Omnibus (GEO) datasets

GEO expression datasets (GSE80617, GSE106336, and GSE140077) were downloaded from National Center for Biotechnology Information (NCBI) website (https://www.ncbi.nlm.nih.gov/). TCGA bladder cancer dataset was downloaded from TCGA official website (https://portal.gdc.cancer.gov).

### Pathway and process enrichment analysis

Pathway and process enrichment analysis was carried out in *Metascape* (http://metascape.org/gp/index.html) with module sources including GO process and KEGG pathway 20. P-values are calculated based on accumulative hypergeometric distribution. Protein-protein interaction networks of the up- or down-regulated proteins were constructed using *String 11.0* (https://string-db.org/).

### Gene set enrichment analysis (GSEA)

GSEA analysis was conducted on TCGA bladder cancer dataset as described previously 21. Gene expression profile of NIBAN1-high (n=202) and NIBAN1-low bladder cancer (n=203) were compared based on enrichment of KEGG pathway signatures. The nominal P value (NOM P-val) and normalized enrichment score (NES) are indicated in the bubble chart. A nominal P value of <0.05 was considered significant.

### Animal study

T24GR cells (2 × 10^6^) with or without NIBAN1 knockdown were subcutaneously injected into the left flank of each nude mice (n=5). Inoculated tumors were allowed to establish for 1 week before initiating gemcitabine treatments. PBS was used as a treatment control. Gemcitabine was intraperitoneally administered (10 mg/kg) once every 2 days 22. Subcutaneous tumors were measured every week. Tumor volume was calculated by the following formula: mm^3^= [length (mm)] × [width (mm)]^2^/2. Mice were sacrificed 4 weeks after tumor inoculation, and the subcutaneous tumors were removed, washed by PBS, and weighted. The expression of NIBAN1, p-FAK, Ki-67, and cleaved caspase-3 in xenograft tissues was evaluated by immunohistochemistry. The immunopositivity of cleaved caspase-3 and Ki-67 in each group was calculated in* six* random, high-power fields.

### Statistical analysis

SPSS 16.0 (SPSS Inc, Chicago, IL, USA) was used for statistical analysis. Error bars throughout the figures indicate standard deviation. Wilcoxon rank sum* test* was used to compare the proteomic data of paired primary/recurrent bladder cancer specimens. The Student's t test (two tailed unpaired) was used to compare means of two groups. One-way ANOVA followed by Tukey's multiple comparisons test was used to compare means of three or more groups. Mann-Whitney test was used to compare the expression of NIBAN1 and p-FAK (Y397) in primary and post-IGC recurrent bladder cancer. The correlation between NIBAN1 and p-FAK (Y397) expression was evaluated by Spearman rank correlation analysis. P < 0.05 was considered significant in all of the tests.

## Results

### Label-free proteomics analysis identifies recurrence-related proteomic profiles in paired primary/recurrent NMIBCs

Before proteomics analyses, FFPE sections of paired primary/post-IGC recurrent tumors were stained with hematoxylin and eosin and examined by a pathologist to localize the cancerous lesions (**Fig. 1A**). Paired primary/post-IGC recurrent NMIBC specimens from a total of eight patients were used for label-free quantitative proteomics analysis; the workflow is illustrated in **Figure 1B**. Collectively, we detected 51082 unique peptides and 6075 proteins, among which 4983 proteins were successfully quantified (**Fig. 1C**). Our label-free quantitative proteomics analysis showed good protein mass distribution and protein sequence coverage (**Fig. 1D, Supplementary Fig. S1A**). In addition, label-free proteomics detection was reproducible, with a mean relative standard deviation less than 0.4% for primary/recurrent NMIBC specimens (**Supplementary Fig. S1B**). The heatmap of Pearson correlation coefficients between primary and recurrent NMIBCs is shown in **Supplementary Figure S1C**. A volcano plot [-log_10_ (P value) vs. log_2_ (fold change [FC])] was plotted to graphically represent the proteomic changes between primary and recurrent NMIBC specimens (**Fig. 1E**). Among the 4983 quantifiable proteins, criteria were set to identify significantly differentially expressed proteins (recurrent vs. primary, Wilcoxon rank-sum test P < 0.05; FC ≥ 1.5 or FC ≤ 0.67). The number of upregulated or downregulated proteins in recurrent versus primary comparison was 105 and 113, respectively (**Fig. 1E, Table S2**). Several upregulated genes (LRP1, SERPINA1, HRG, ILK, CAV1, and LAMA4) identified in our study are involved in GEM resistance in human cancer 23-28.

### Multiple biological processes/signaling pathways might be involved in NMIBC recurrence after IGC

**Figure 2:**
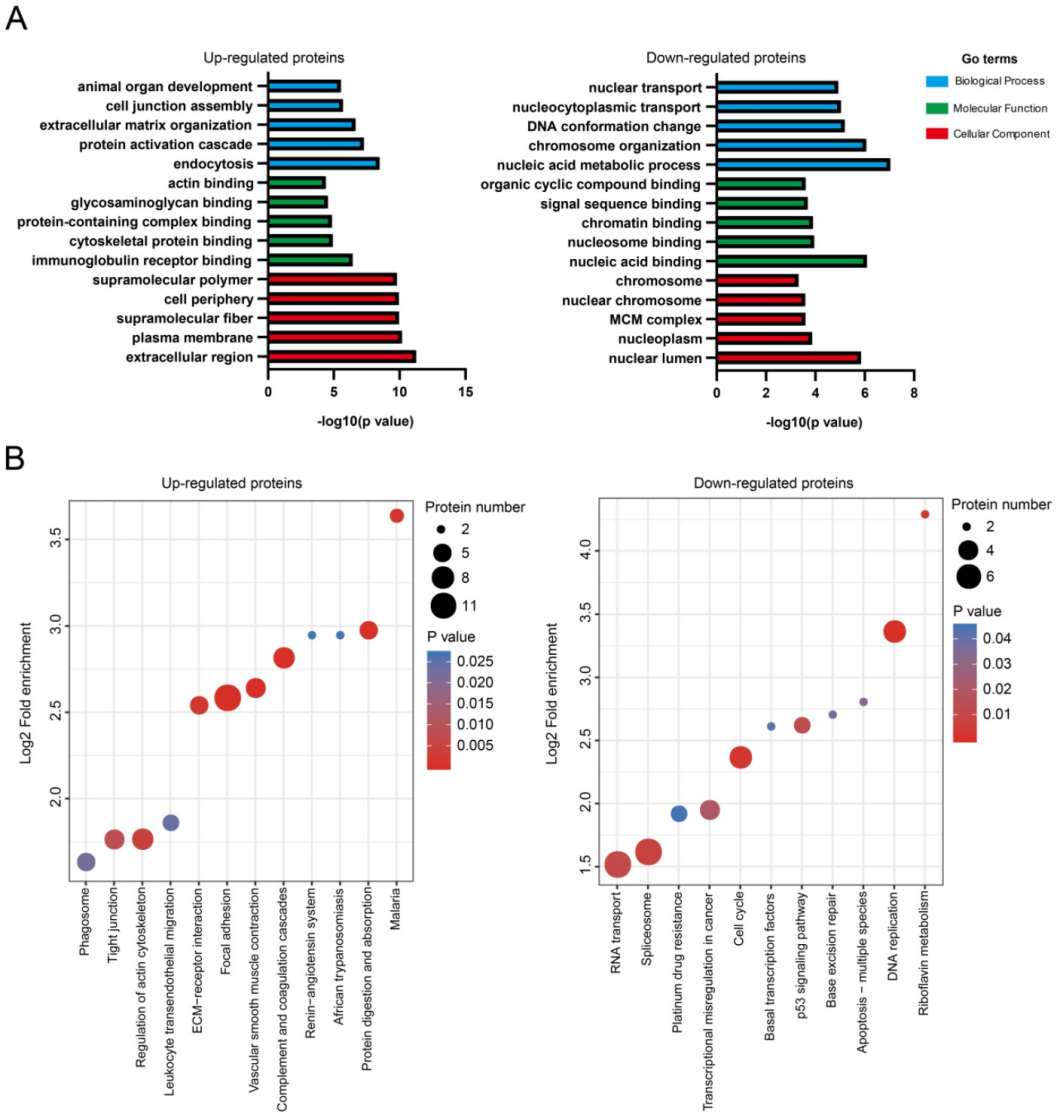
** Multiple biological processes/signaling pathways might be involved in NMIBC recurrence after IGC. A**. Enrichment of GO for upregulated or downregulated proteins. **B**. Enrichment of KEGG pathways for upregulated or downregulated proteins.

Differentially expressed proteins were subjected to bioinformatics analysis using the Gene Ontology (GO) and Kyoto Encyclopedia of Genes and Genomes (KEGG) pathway databases. For upregulated proteins (n = 105), the GO terms in biological process (endocytosis, protein activation cascade, extracellular matrix organization, cell junction assembly, and animal organ development), molecular function (immunoglobulin receptor binding, cytoskeletal protein binding, protein-containing complex binding, glycosaminoglycan binding, and actin binding), and cellular components (extracellular region, plasma membrane, supramolecular fiber, cell periphery and supramolecular polymer) were highly enriched (**Fig. 2A**). For the downregulated proteins (n = 113), the GO terms in biological process (nucleic acid metabolic process, chromosome organization, DNA conformation change, nucleocytoplasmic transport, and nuclear transport), molecular function (nucleic acid binding, nucleosome binding, chromatin binding, signal sequence binding, and organic cyclic compound binding), and cellular components (nuclear lumen, nucleoplasm, minichromosome maintenance protein complex, nuclear chromosome, and chromosome) were highly enriched (**Fig. 2A**).

KEGG pathways including focal adhesion (ITGA5, COL6A1, COL6A2, COL6A3, ACTN1, FLNA, CAV1, ILK, MYLK, LAMA4, PARVA, TLN1), complement and coagulation cascades (F13A1, CFD, F12, SERPINC1, SERPINA1, A2M), and vascular smooth muscle contraction (AGT, MYH11, MYL6, ACTA2, ACTG2, MYLK) were highly enriched for the upregulated proteins (**Fig. 2B**). The downregulated proteins was shown to enrich KEGG pathways including DNA replication (PCNA, MCM3, MCM7, FEN1, MCM2), cell cycle (PCNA, MCM3, MCM7, MCM2, ATM), and spliceosome (SRSF10, SNRPF, SF3B4, USP39, RBMXL1, RBM22, WBP11) (**Fig. 2B**). The graphical network map of the focal adhesion pathway is shown in **Figure S2**.

**Figure 3:**
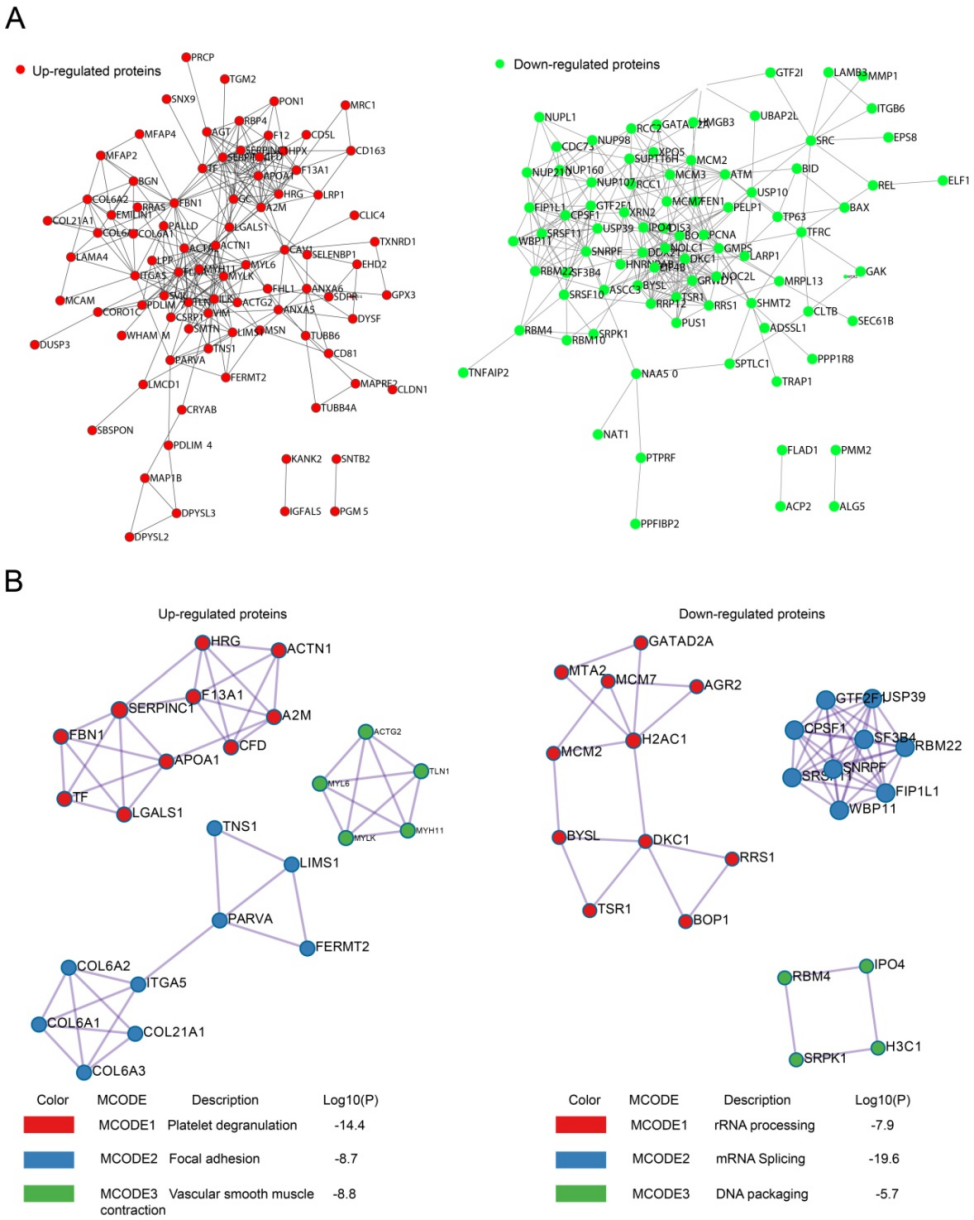
** PPI for upregulated or downregulated proteins. A.** PPI networks encoded by upregulated or downregulated proteins. **B**. Top three MCODE modules for upregulated or downregulated proteins.

Protein-protein interaction (PPI) analysis with String 11.0 revealed that recurrence-related proteins could interact with each other to constitute a large PPI networks, which was composed of 98 nodes and 282 edges for upregulated proteins and 110 nodes and 299 edges for downregulated proteins (**Fig. 3A**). We also searched for hub genes among upregulated proteins using the cytoHubba plugin of Cytoscape (bottleneck method). The top 10 hub genes in upregulated proteins were ACTN1, FLNA, FBN1, AHSG, SERPINA1, A2M, APOA1, ACTA2, TLN1, and MYLK, with scores ranging from 23 to 14.

The PPI network was further analyzed by the Molecular Complex Detection (MCODE) plugin. The top three most significant modules were identified using GO, KEGG, and REACTOME annotations. For the upregulated proteins, MCODE1 was mainly involved in platelet degranulation, whereas MCODE2 and MCODE 3 were associated with focal adhesion and vascular smooth muscle contraction, respectively (**Fig. 3B**). For the downregulated proteins, MCODE1 was mainly involved in RNA processing, whereas MCODE2 and MCODE 3 were associated with mRNA splicing and DNA packaging, respectively (**Fig. 3B**).

### NIBAN1 might be a novel regulator of GEM resistance in bladder cancer

**Figure 4:**
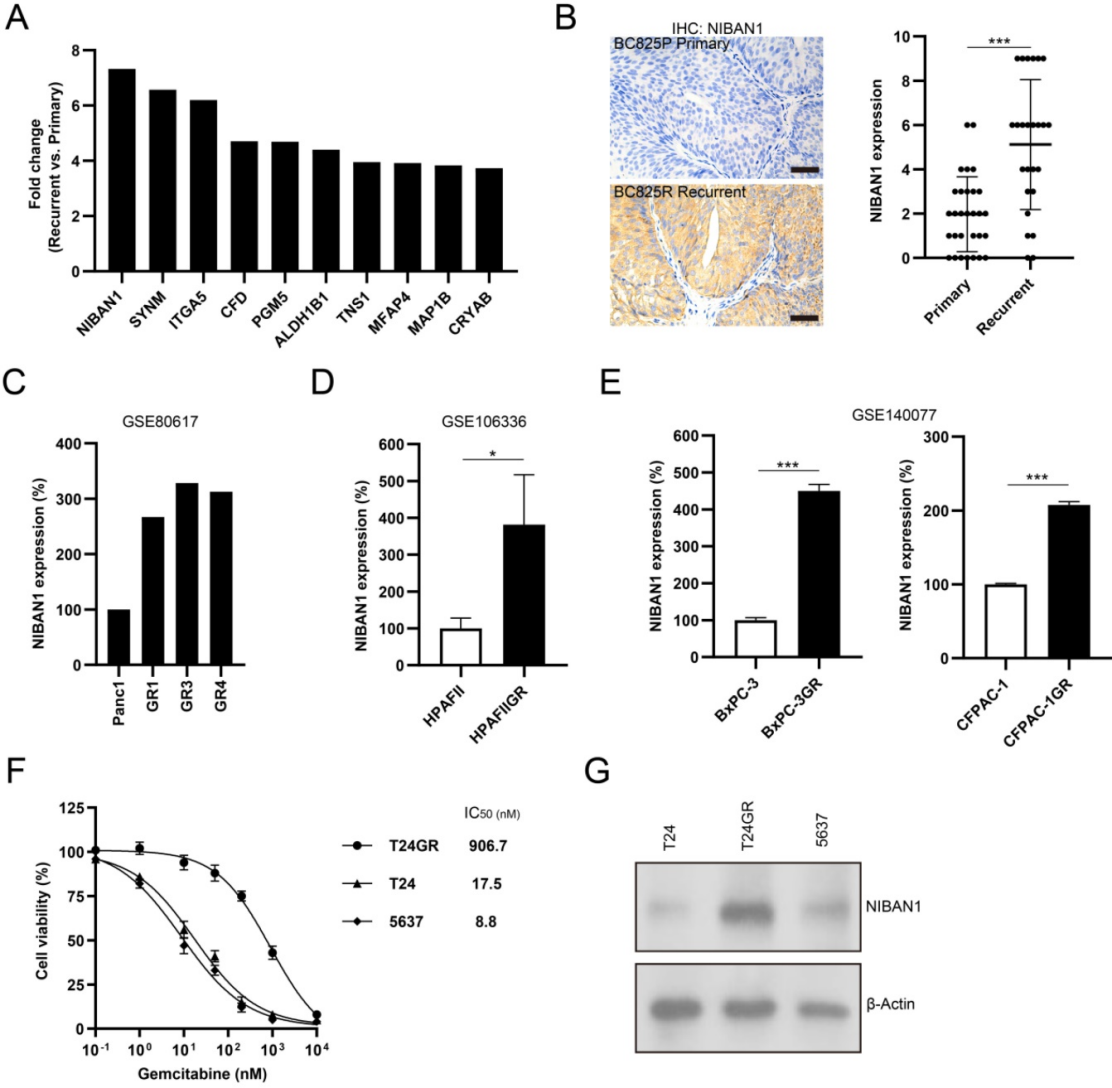
** NIBAN1 might be a novel regulator of GEM resistance in bladder cancer. A**. Top 10 upregulated proteins in recurrent NMIBCs. **B**. Expression of NIBAN1 in primary (n = 30) and post-IGC recurrent (n = 25) NMIBCs. ***P < 0.001. Bars: 100 μm. **C-E**. Expression of NIBAN1 in a number of GEM-resistant sublines and their parental cell lines. *P < 0.05; ***P < 0.001. **F.** T24GR is highly resistant to GEM compared to T24 and 5637. **G.** Expression of NIBAN1 in GEM-sensitive and GEM-resistant bladder cancer cells. β-actin was used as a loading control.

The top 10 upregulated proteins in recurrent NMIBC are shown in **Figure 4A**. NIBAN1, also known as FAM129A, was identified as the top highly expressed protein in post-IGC recurrent NMIBCs. The mass spectrum of the identified NIBAN1 unique peptide (VLTSEDEYNLLSDR) is shown in **Supplementary Figure S3**. Next, we examined the expression of NIBAN1 in chemonaive primary (n = 30) and post-IGC recurrent (n = 25) NMIBCs. The protein expression of NIBAN was greatly increased in post-IGC recurrent NMIBCs compared to chemonaive primary NMIBCs (**Fig. 4B**). To confirm the involvement of NIBAN1 in GEM resistance, we analyzed the expression of NIBAN1 in several GEM resistance-related Gene Expression Ominbus (GEO) datasets. Analysis of GEO datasets (GSE80617, GSE106336, and GSE140077) revealed increased NIBAN1 expression in GEM-resistant Panc1, HPAFII, BxPC-3, and CFPAC-1 sublines compared to their parental cell lines (**Fig. 4C-E**). Therefore, NIBAN1 might be involved in the regulation of GEM resistance, which needs to be functionally validated in bladder cancer cell models. Cell Counting Kit-8 cytotoxicity analysis showed that GEM-resistant T24GR exhibited a considerably higher GEM IC_50_ value than GEM-sensitive T24 and 5637 cells (**Fig. 4F**). Consistently, NIBAN1 expression was markedly higher in T24GR than in T24 and 5637 (**Fig. 4G**). Therefore, NIBAN1 might be a novel regulator of GEM resistance in bladder cancer.

### Knockdown of NIBAN1 sensitizes GEM-resistant bladder cancer cells to GEM treatment

**Figure 5:**
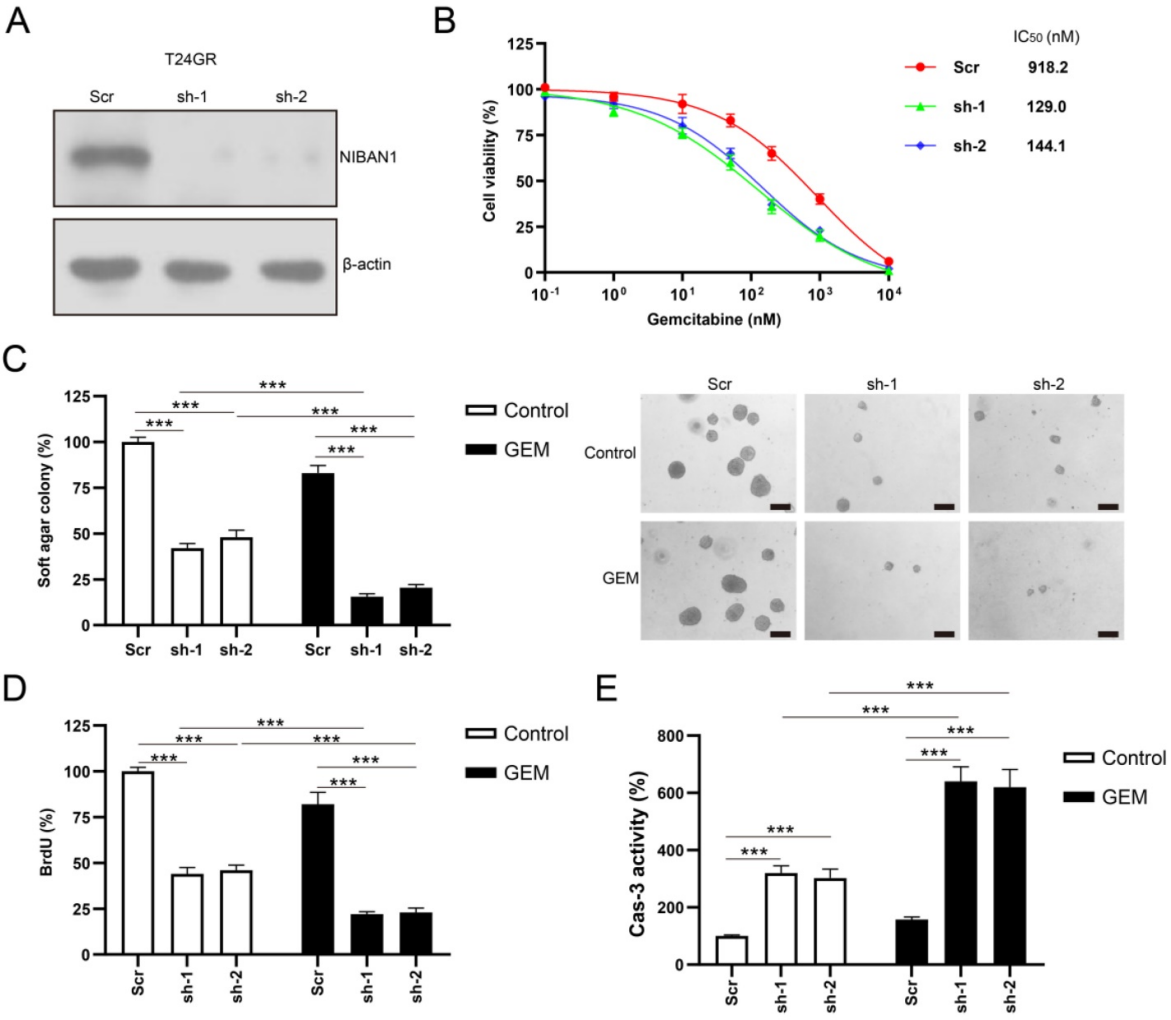
**Knockdown of NIBAN1 sensitizes GEM-resistant bladder cancer cells to GEM treatment. A**. Lentivirus-mediated shRNA knockdown markedly attenuated NIBAN1 expression in T24GR cells. β-actin was used as a loading control. **B**. Knockdown of NIBAN1 sensitized T24GR cells to GEM treatment. **C**. Knockdown of NIBAN1 impaired soft agar clonogenesis after GEM treatment (100 nM) in T24GR cells. PBS was used as a treatment control. n = 4, ***P < 0.001. Bars: 200 μm. **D**. Knockdown of NIBAN1 reduced BrdU incorporation following GEM treatment (100 nM) for 48 h in T24GR cells. PBS was used as a treatment control. n = 4, ***P < 0.001. **E.** Knockdown of NIBAN1 increased caspase-3 activity following GEM treatment (100 nM) for 48 h in T24GR cells. PBS was used as a treatment control. n = 4, ***P < 0.001.

The protein expression of NIBAN1 was markedly reduced by NIBAN1-specific short hairpin RNAs (shRNAs) in T24GR cells** (Fig. 5A)**. The effect of NIBAN1 knockdown on the chemosensitivity of T24GR cells was examined. NIBAN1 knockdown greatly attenuated the chemoresistance to GEM in T24GR cells (**Fig. 5B**). Soft agar clonogenesis of NIBAN1-depleted GTN cells was markedly decreased compared to the scrambled (Scr) control (**Fig. 5C**). NIBAN1 knockdown further reduced soft agar clonogenesis compared to the Scr control after GEM treatment (**Fig. 5C**). Furthermore, knockdown of NIBAN1 markedly reduced BrdU incorporation and increased caspase-3 activity in T24GR cells (**Fig. 5D, E**). Compared with the Scr group, reduced BrdU incorporation and increased caspase-3 activities were also observed in the shNIBAN1 group after GEM treatment (**Fig. 5D, E**).

### Overexpression of NIBAN1 promotes GEM resistance in GEM-sensitive bladder cancer cells

**Figure 6:**
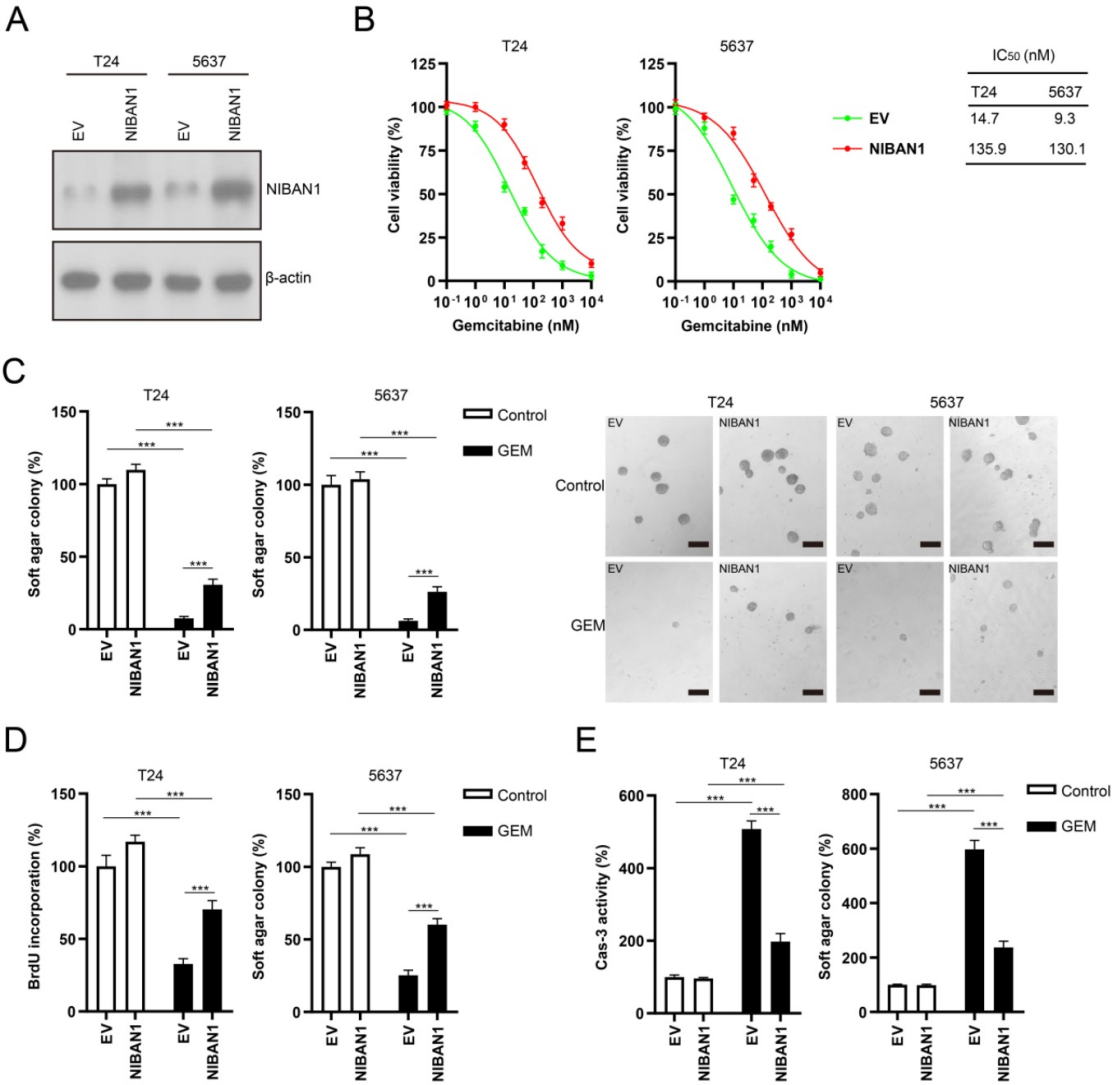
**Overexpression of NIBAN1 promotes GEM resistance in GEM-sensitive bladder cancer cells**. **A**. The expression of NIBAN1 was greatly increased in T24 and 5637 cells. β-actin was used as a loading control. **B**. Overexpression of NIBAN1 promoted chemoresistance to GEM in T24 and 5637 cells. **C**. Overexpression of NIBAN1 rescued soft agar clonogenesis after GEM treatment (10 nM) in T24 and 5637 cells. PBS was used as a treatment control. n = 4, ***P < 0.001. **D.** Overexpression of NIBAN1 greatly restored BrdU incorporation following GEM treatment (10 nM) for 48 h in T24 and 5637 cells. PBS was used as a treatment control. n = 4, ***P < 0.001.** E**. Overexpression of NIBAN1 attenuated caspase-3 activity following GEM treatment (10 nM) for 48 h in T24 and 5637 cells. PBS was used as a treatment control. n = 4, ***P < 0.001.

We also overexpressed NIBAN1 in GEM-sensitive T24 and 5637 cells. The level of NIBAN1 was greatly increased after transfection of the NIBAN1 plasmid in these two cell lines (**Fig. 6A**). The cytotoxic effect of GEM on NIBAN1-expressing T24 and 5637 cells was evaluated. NIBAN1 group exhibited a markedly higher IC_50_ than the empty vector (EV) group after GEM treatment of T24 and 5637 cells (**Fig. 6B**). In addition, overexpression of NIBAN1 also significantly promoted soft agar clonogenesis compared with EV after GEM treatment of T24 and 5637 cells (**Fig. 6C**). Furthermore, the NIBAN1 group exhibited higher BrdU incorporation and lower caspase-3 activities after GEM treatment than the EV group (**Fig. 6D, E**). These results suggest that overexpression of NIBAN1 can promote chemoresistance to GEM in GEM-sensitive bladder cancer cells.

### High NIBAN1 expression is correlated with focal adhesion/focal adhesion kinase signaling activation in bladder cancer

**Figure 7:**
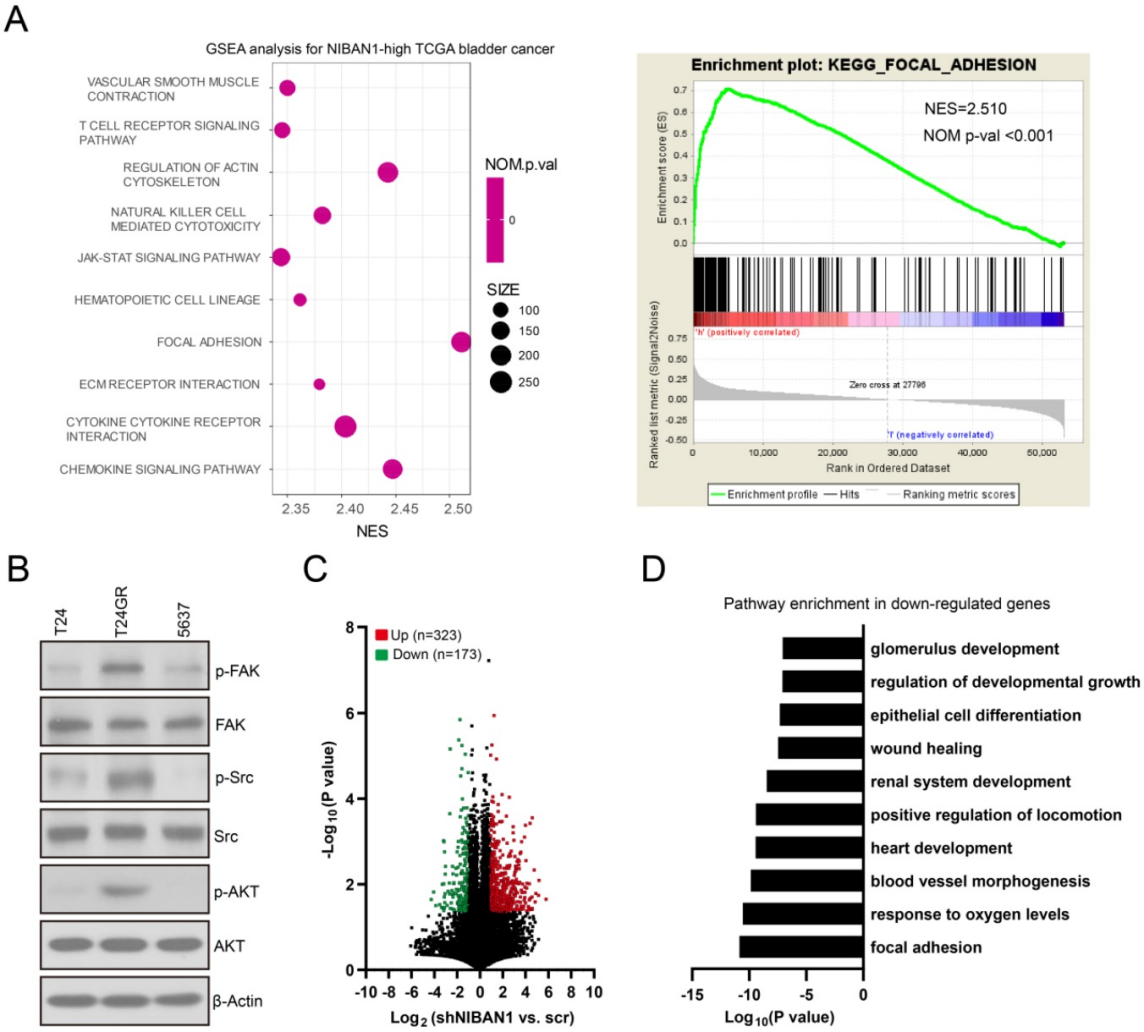
** High NIBAN1 expression correlates with highly enriched focal adhesion signaling in bladder cancer**. **A**. Gene Set Enrichment Analysis of The Cancer Genome Atlas bladder cancer dataset revealed a highly enriched gene signature of focal adhesion pathway in NIBAN1-high bladder cancer. **B**. GEM-resistant cells T24GR exhibited highly activated FAK and its downstream SRC/AKT signaling compared to GEM-sensitive T24 and 5637 cells. β-Actin was used as a loading control. **C.** Differentially expressed genes in T24GR cells with or without NIBAN1 knockdown. **D**. Pathway enrichment analysis was conducted on downregulated genes (n = 173) using GO, KEGG, and Reactome Gene Sets annotations.

We conducted Gene Set Enrichment Analysis on The Cancer Genome Atlas bladder cancer dataset to identify the downstream signaling pathways regulated by NIBAN1. Our analysis revealed that the focal adhesion signaling pathway is the most enriched signaling pathway in NIBAN1-high bladder cancer (**Fig. 7A**). Focal adhesion kinase (FAK) is a pivotal kinase that regulates focal adhesion process in response to environmental signals initiated by growth factors, extracellular matrix, and surrounding mechanical forces, thus promoting cancer cell growth, migration, and survival 29. Consistent with NIBAN1 blot (**Fig. 4G**), we observed highly activated FAK (FAK pY397) and its downstream SRC/AKT signaling in GEM-resistant T24GR cells compared to GEM-sensitive T24 and 5637 cells (**Fig. 7B**). Next, we compared the transcriptomic profiles of T24GR cells with or without NIBAN1 knockdown. The number of upregulated (Log_2_FC ≥ 1, P < 0.05) or downregulated (Log_2_FC ≤ -1, P<0.05) genes in shNIBAN1 versus the Scr group was 323 and 173, respectively (**Fig. 7C, Table S3**). Pathway enrichment analysis of downregulated genes (n = 173) using GO, KEGG, and Reactome Gene Sets annotations showed that knockdown of NIBAN1 attenuated the activity of several signaling pathways including focal adhesion, response to oxygen levels, and blood vessel morphogenesis (**Fig. 7D**)*.* These findings might connect the possible function of NIBAN1 with focal adhesion/FAK signaling activation in bladder cancer.

### NIBAN1 regulates FAK and its downstream signaling activity in bladder cancer cell lines

**Figure 8:**
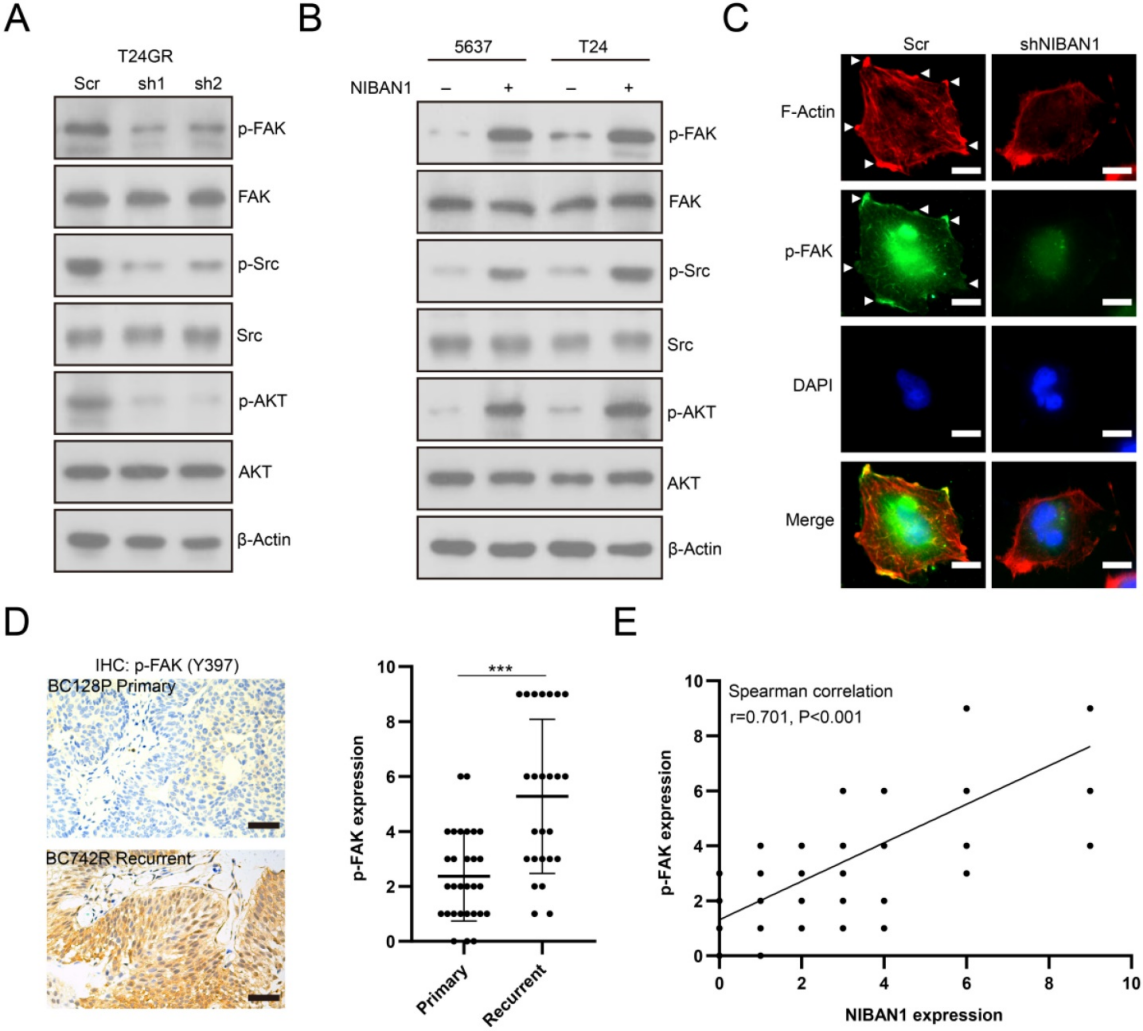
NIBAN1 regulates focal adhesions by modulating FAK signaling activation in bladder cancer cells. **A**. Knockdown of NIBAN1 attenuated FAK and its downstream SRC/AKT signaling activation in T24GR cells. β-Actin was used as a loading control. **B.** Overexpression of NIBAN1 promoted FAK and its downstream SRC/AKT signaling activation in T24 and 5637 cells. β-Actin was used as a loading control. **C**. Immunofluorescence staining of F-actin and p-FAK in T24GR cells with or without NIBAN1 knockdown. White triangles indicate the protrusions. Bars: 10 μm. **D**. Expression of p-FAK (Y397) in primary (n = 30) and post-IGC recurrent (n = 25) NMIBCs. ***P < 0.001. Bars: 100 μm. **E**. Correlated expression of NIBAN1 and p-FAK (Y397) in NMIBC specimens.

We investigated the effect of NIBAN1 on FAK signaling activity in bladder cancer cell models. Knockdown of NIBAN1 greatly reduced the level of phosphorylated FAK (p-FAK) and its downstream p-SRC and p-AKT in T24GR cells (**Fig. 8A**). By contrast, overexpression of NIBAN1 activated FAK and its downstream SRC/AKT signaling in T24 and 5637 cells** (Fig. 8B)**. In addition, immunofluorescence staining was conducted to evaluate the effect of NIBAN1 knockdown on focal adhesions. As shown in **Figure 8C**, knockdown of NIBAN1 considerably reduced the formation of long F-actin stress fibers and protrusions/lamellipodia edges. Consistently, knockdown of NIBAN1 also reduced p-FAK expression and p-FAK localization in the edge and protrusions (**Fig. 8C**). These results suggest that NIBAN1 might regulate the formation of focal adhesions by activating the FAK signaling pathway. The expression of p-FAK was examined in chemonaive primary (n = 30) and post-IGC recurrent (n = 25) NMIBCs. Increased p-FAK expression was observed in post-IGC recurrent NMIBCs compared to chemonaive primary NMIBCs (**Fig. 8D**). Correlated expression of NIBAN1 and p-FAK was seen in these NMIBC specimens (**Fig. 8E**).

### Knockdown of FAK sensitizes GEM-resistant bladder cancer cells to GEM treatment

**Figure 9:**
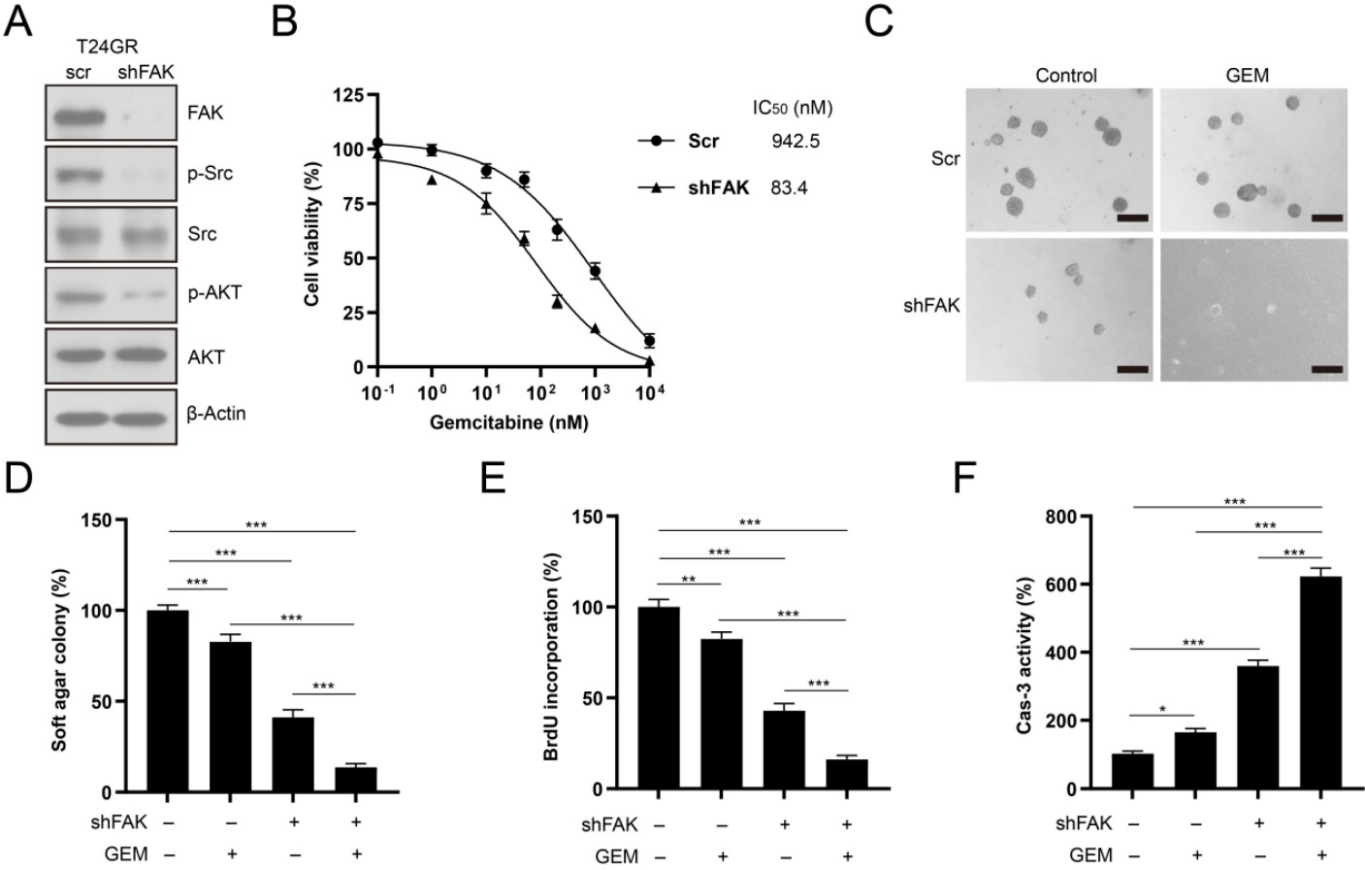
Knockdown of FAK sensitized T24GR cells to GEM treatment. **A**. Knockdown of FAK attenuated its downstream SRC/AKT signaling activation in T24GR cells. β-Actin was used as a loading control. **B**. Knockdown of FAK sensitized T24GR cells to GEM treatment. **C, D**. Knockdown of FAK impaired soft agar clonogenesis after GEM treatment in T24GR cells. PBS was used as a treatment control. n=4, ***P<0.001. Bars: 250 μm. **E**. Knockdown of FAK reduced BrdU incorporation following GEM treatment for 48 hours in T24GR cells. PBS was used as a treatment control. n=4, **P<0.01; ***P<0.001. **F.** Knockdown of FAK increased caspase-3 activity following GEM treatment for 48 hours in T24GR cells. PBS was used as a treatment control. n=4, *P<0.05; ***P<0.001.

We depleted FAK expression in T24GR cells to confirm the involvement of FAK signaling in the development of GEM resistance. FAK depletion abolished its downstream SRC/AKT signaling activity in T24GR cells (**Fig. 9A**). In addition, knockdown of FAK reduced the GEM IC_50_ value in T24GR cells (**Fig. 9B**). Furthermore, depletion of FAK also markedly reduced soft agar clonogenesis/BrdU incorporation and increased caspase-3 activity after GEM treatment in T24GR cells (**Fig. 9C-F**). Therefore, NIBAN1 might regulate FAK signaling activation to promote GEM resistance in bladder cancer cells.

### Knockdown of NIBAN1 sensitizes GEM-resistant T24GR cells to GEM treatment in a murine xenograft model

**Figure 10:**
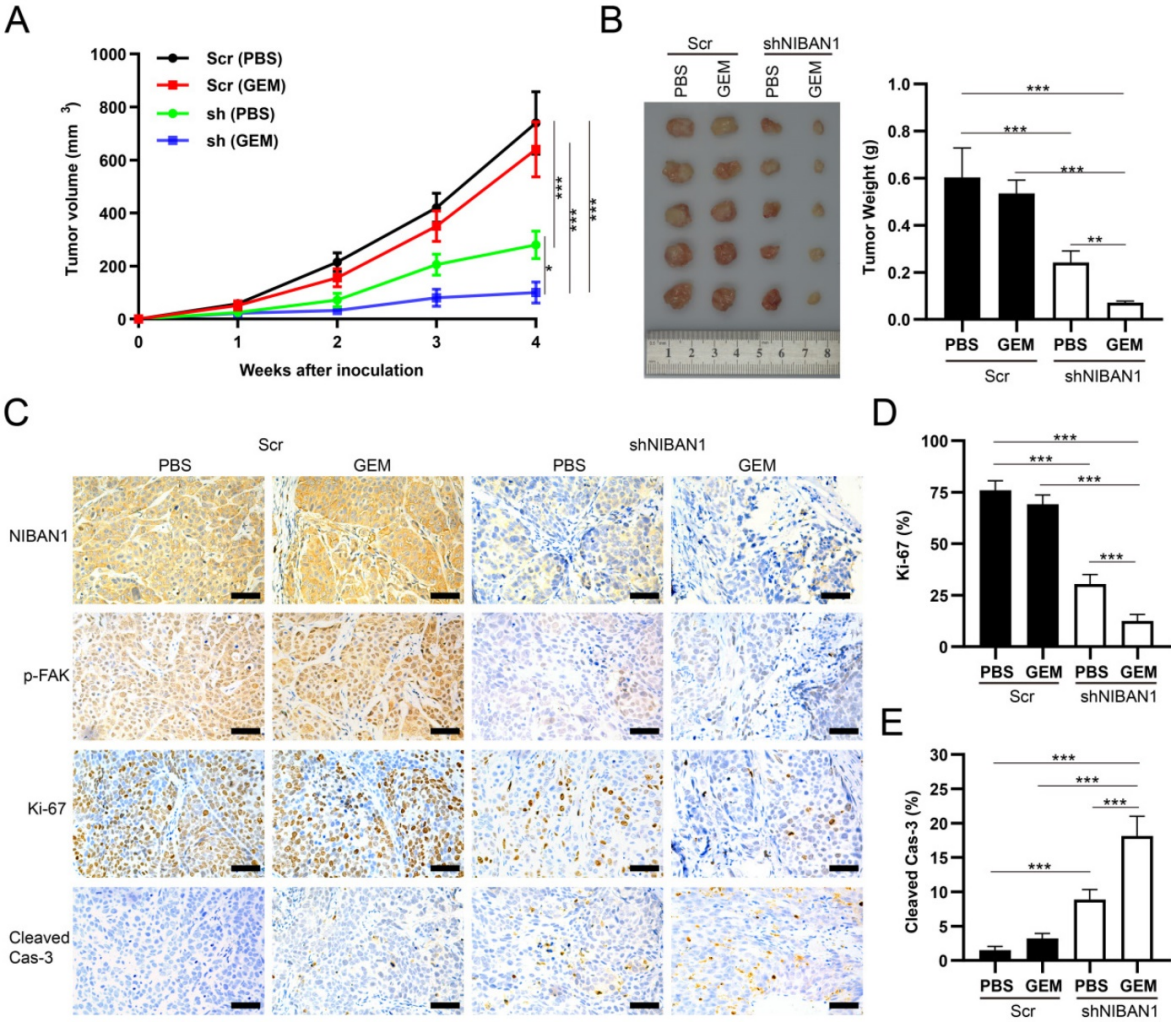
NIBAN1 knockdown sensitizes GEM-resistant bladder cancer cells to GEM treatment. **A**. Tumor volume of subcutaneous T24GR xenografts (Scr or shNIBAN1) after GEM treatment for indicated time. PBS was used as a treatment control. n=5, *P<0.05; ***P<0.001. **B**. NIBAN1 knockdown markedly reduced the weight of T24GR xenografts after GEM treatment. PBS was used as a treatment control. n=5, **P<0.01; ***P<0.001. **C**. IHC staining of T24GR xenografts (Scr or shNIBAN1) after GEM treatment. PBS was used as a treatment control. Bars: 100 μm. **D, E**. Ki-67 and cleaved caspase-3 positivity in T24GR xenografts (Scr or shNIBAN1) after GEM treatment. PBS was used as a treatment control. n=6, ***P<0.001.

The *in vivo* effect of NIBAN1 knockdown on the chemosensitivity of T24GR cells was evaluated in a murine xenograft model. GEM treatment exhibited a mild inhibitory effect on tumor growth compared to phosphate-buffered saline (PBS) in T24GR Scr tumors. However, NIBAN1 knockdown effectively suppressed the tumor growth after GEM treatment compared with Scr (PBS), Scr (GEM), or shNIBAN (PBS) group (**Fig. 10A**). Similarly, the tumor weights in the shNIBAN1 group were maximally reduced after GEM treatment compared with the other three groups (**Fig. 10B**). Immunohistochemistry staining showed that knockdown of NIBAN1 markedly attenuated p-FAK expression in xenograft tissues with or without GEM treatment (**Fig. 10C**). In addition, GEM treatment maximally reduced Ki-67 positivity and increased cleaved caspase-3 labeling in the shNIBAN1 group compared to the other three groups (**Fig. 10C-E**). Taken together, these findings confirm the role of NIBAN1 as a potential regulator of GEM resistance in bladder cancer *in vivo*.

## Discussion

Although postoperative IGC is a promising adjuvant therapy to reduce the risk of recurrence of NMIBC, a considerable fraction of patients still experience cancer recurrence within 2 years. One of the possible factors contributing to cancer recurrence is the development of chemoresistance after IGC 30. In this study, we employed a label-free quantitative proteomics approach to discover novel regulators of GEM resistance in bladder cancer. To our knowledge, this is the first study to compare the proteomics profiles of primary and post-IGC recurrent NMIBC. A number of signaling pathways (e.g., focal adhesion, complement and coagulation cascades, and vascular smooth muscle contraction) were found to be highly activated in post-IGC recurrent NMIBC. Therefore, multiple molecular mechanisms might contribute to the development of cancer recurrence/GEM resistance in NMIBC. Elucidating these mechanisms may help develop more effective therapeutic strategies to reduce the risk of NMIBC recurrence in the future.

Recent findings have implicated NIBAN1 in the cellular response to stress. Sun *et al.* 26 showed that NIBAN1 is involved in the endoplasmic reticulum (ER) stress response and can antagonize cell death signaling by regulating translation. In thyroid carcinoma, the expression of NIBAN1 is highly elevated under nutrient/growth factor deprivation 31. In addition, NIBAN1 can also attenuate angiotensin II- and ER stress-induced apoptosis in renal tubular epithelial cells 32. Recently, Cevik *et al*. 29 revealed that NIBAN1 might be involved in the cellular response to stress conditions (linoleic acid, hydrogen peroxide, and ethanol) in adipocytes. Because chemotherapeutic drugs can also act as stressors, it is reasonable to hypothesize that NIBAN might also exert cytoprotective/anti-apoptosis functions in response to GEM treatment in bladder cancer. Consistent with this hypothesis, we observed highly increased expression of NIBAN1 in GEM-resistant cancer cell lines and in post-IGC recurrent NMIBC specimens. Furthermore, our studies in *in vitro* cell line models showed that manipulation of NIBAN1 expression affected the chemosensitivity to GEM in bladder cancer cell models, suggesting that NIBAN1 might serve as a potential regulator of GEM resistance in bladder cancer. Understanding the molecular pathways modulated by NIBAN1 may contribute to improvements in the current therapeutic regimens for NMIBC.

Focal adhesion signaling pathways integrate multiple signaling molecules including integrins, growth factor receptors, and protein kinases/phosphates that regulate cancer cell survival, proliferation, and motility 33. FAK acts as a pivotal node of multiple signaling pathways coupling extracellular and cytosolic signals at focal adhesions 34. Recent studies have revealed an emerging role of FAK in promoting chemoresistance to taxane and platinum-based therapy in ovarian and other cancers. Kang *et al*. 32 showed that FAK can regulate Y-box binding protein 1-mediated paclitaxel resistance in ovarian cancer; activated FAK is found in doxorubicin-resistant MCF-7/Dox cells compared with parental MCF-7 cells 35; FAK overexpression can upregulate alcohol dehydrogenase and X-linked inhibitor of apoptosis protein activity in platinum-resistant ovarian cancer cells 36. However, whether FAK signaling is involved in the regulation of GEM resistance in bladder cancer remains unknown. Herein, our findings revealed that focal adhesion/FAK signaling is highly activated in post-IGC recurrent NMIBC. In addition, we showed that NIBAN1 could regulate FAK and its downstream SRC/AKT signaling activation in bladder cancer cell line models. Similar to NIBAN1 findings, knockdown of FAK also markedly attenuated GEM-resistant phenotypes in bladder cancer cells. Our *in vivo* studies demonstrated that knockdown of NIBAN1 disrupted FAK signaling and sensitized GEM-resistant bladder cancer cells to GEM treatment. Therefore, NIBAN1 might regulate FAK signaling activation to promote GEM resistance in bladder cancer, and targeting NIBAN1/FAK signaling could potentially sensitize bladder cancer cells to GEM chemotherapy.

In summary, our study characterized the proteomics profiles associated with cancer recurrence after IGC in NMIBC. We showed that NIBAN1 might modulate activation of the FAK signaling pathway to promote GEM resistance in bladder cancer cells. Our findings highlight the potential role of NIBAN1/FAK signaling in the regulation of GEM resistance in NMIBC, which might have potential translational value in designing experimental therapeutics to treat NMIBC.

## Supplementary Material

Supplementary methods, figures and tables.
