# Supplementary material for: Niban apoptosis regulator 1 promotes gemcitabine resistance by activating the focal adhesion kinase signaling pathway in bladder cancer
Source: J Cancer. 2022 Jan 9;13(4):1103–18. doi: 10.7150/jca.66248 (PMC8899363; doi:10.7150/jca.66248)
Supplement: Supplementary file 1 — Supplementary methods, figures and tables. [file jcav13p1103s1.pdf]

Supplementary methods

1. Methods for label free proteomic analysis

1.1 Protein extraction and trypsin digestion

For each FFPE sample, 3-4 sections 15-20 μm each were obtained and combined in a 2 ml eppendorf tube for further processing. This included, deparaffinization through three incubations in xylene (first two for 5 min each and the last for 1 min), each one followed by centrifugation for 3 min at 13,000 rpm at room temperature (RT). The sections were then rehydrated through a series of ethanol and distilled water washes (100% ethanol for 2 min, 95% ethanol for 1 min, 70% ethanol for 1 min, distilled water for 1 min), each one followed by centrifugation for 3 min at 13,000 rpm, at RT. Upon rehydration, the tissue pellets were left to air dry for 30 min at RT. The samples were resuspended in 200 μl of FASP buffer (pH ~ 8), containing 100 mM Tris-HCl, 4 % SDS, 100 mM DTE. For homogenization, 0.9 - 2.0 mm stainless steel beads were added to the samples and placed at the bullet blender homogenizer in 2 sequential steps: 5 min in at speed 12 and 3 min in at speed 10. The homogenates were sonicated for 3 cycles of 5 seconds each using a tip sonicator (36% power used), followed by 1 h of heating at 90 °C on a heating block. Then, the extracts were centrifuged for 10 min at 13,000 rpm at RT and the supernatants (~170 μl) were transferred in to new 1.5 ml eppendorf tubes. Trypsin was added at 1:50 trypsin-to-protein mass ratio for digestion overnight at 37 °C. The hydrolysates was reduced with 5 mM dithiothreitol for 30 min in 56 °C and alkylated with 11 mM iodoacetamide for 15 min at room temperature in darkness. Then the tryptic peptides were desalted with SPE column and dried by vacuum centrifuging.

1.2 LC-MS/MS Analysis

The tryptic peptides were dissolved in solvent A (0.1% formic acid, 2% acetonitrile/ in water), directly loaded onto a home-made reversed-phase analytical column (25-cm length, 100 μm i.d.). Peptides were separated with a gradient from 6%

to 24% solvent B (0.1% formic acid in acetonitrile) over 70 min, 24% to 35% in 14 min and climbing to 80% in 3 min then holding at 80% for the last 3 min, all at a constant flow rate of 450 nL/min on a nanoElute UHPLC system (Bruker Daltonics). The peptides were subjected to Capillary source followed by the timsTOF Pro (Bruker Daltonics) mass spectrometry. The electrospray voltage applied was 1.70 kV. Precursors and fragments were analyzed at the TOF detector, with a MS/MS scan range from 100 to 1700 m/z. The timsTOF Pro was operated in parallel accumulation serial fragmentation (PASEF) mode. Precursors with charge states 0 to 5 were selected for fragmentation, and 10 PASEF-MS/MS scans were acquired per cycle. The dynamic exclusion was set to 30 s.

1.3 Database Search

The resulting MS/MS data were processed using MaxQuant search engine (v.1.6.15.0). Tandem mass spectra were searched against the Homo Sapiens database (SwissProt, 20395 entries) concatenated with reverse decoy database. Trypsin/P was specified as cleavage enzyme allowing up to 2 missing cleavages. The mass tolerance for precursor ions was set as 20 ppm in First search and 20 ppm in Main search, and the mass tolerance for fragment ions was set as 0.02 Da. Carbamidomethyl on Cys was specified as fixed modification, and acetylation on protein N-terminal and oxidation on Met were specified as variable modifications. FDR was adjusted to < 1%.

2. Methods for lentivirus packaging and infection

Lentiviral expression Lv105 plasmids encoding empty vector (EV) or NIBAN1 were obtained from Genecopoeia Inc. Lentiviral shRNA plasmids encoding scramble control, shNIBAN1, and shFAK/PTK2 were also provided by Genecopoeia Inc. The sequences for the shRNAs and control shRNA used in this study are listed as follows: NIBAN1 shRNAs: sh-1: CCAAGCC ACCATTGGCGCCTG; sh-2: CAGCCCTTTGTGGTCCTGCCC. Scramble control: CCTAAGGTTAAGTCGCCCTCG. These lentiviral plasmids were cotransfected into 293FT cells with a Lentiviral Packaging Kit (Genecopoeia) to obtain recombinant

lentiviruses.

For the generation of stable cell lines, bladder cancer cells were cultured in 60mm culture dishes overnight. The collected recombinant lentiviruses supplemented with 8 μg/ml polybrene were added to the adherent cells. After 48 h of viral infection, the supernatant was replaced with fresh medium containing 1 μg/ ml puromycin, and incubated for 1 week.

Supplementary figures

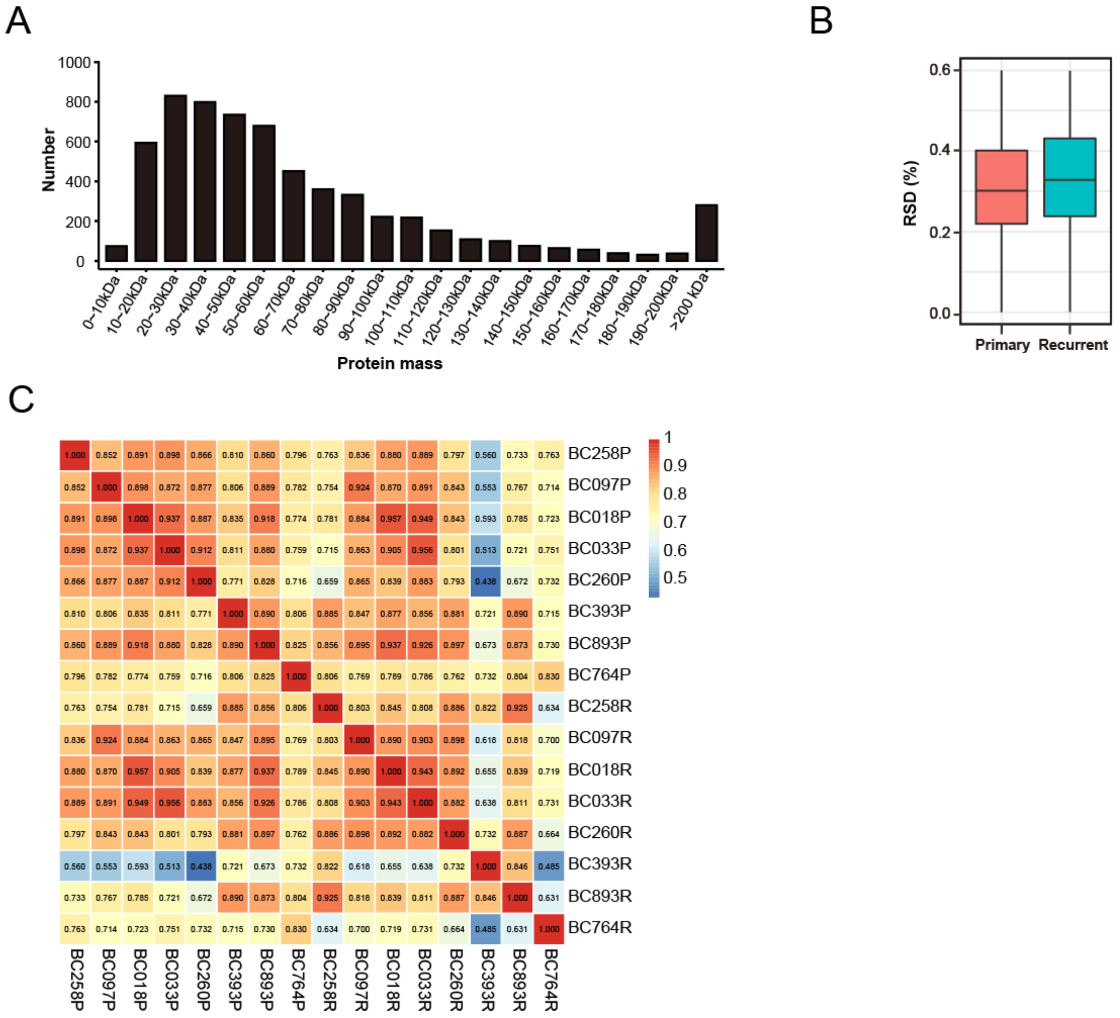

Figure S1: **A**. Distribution of protein mass of identified proteins. **B**. Relative standard deviation (RSD) of proteomic data of primary/recurrent NMIBCs. **C**. The heatmap of Pearson correlation coefficients between paired primary and recurrent NMIBCs

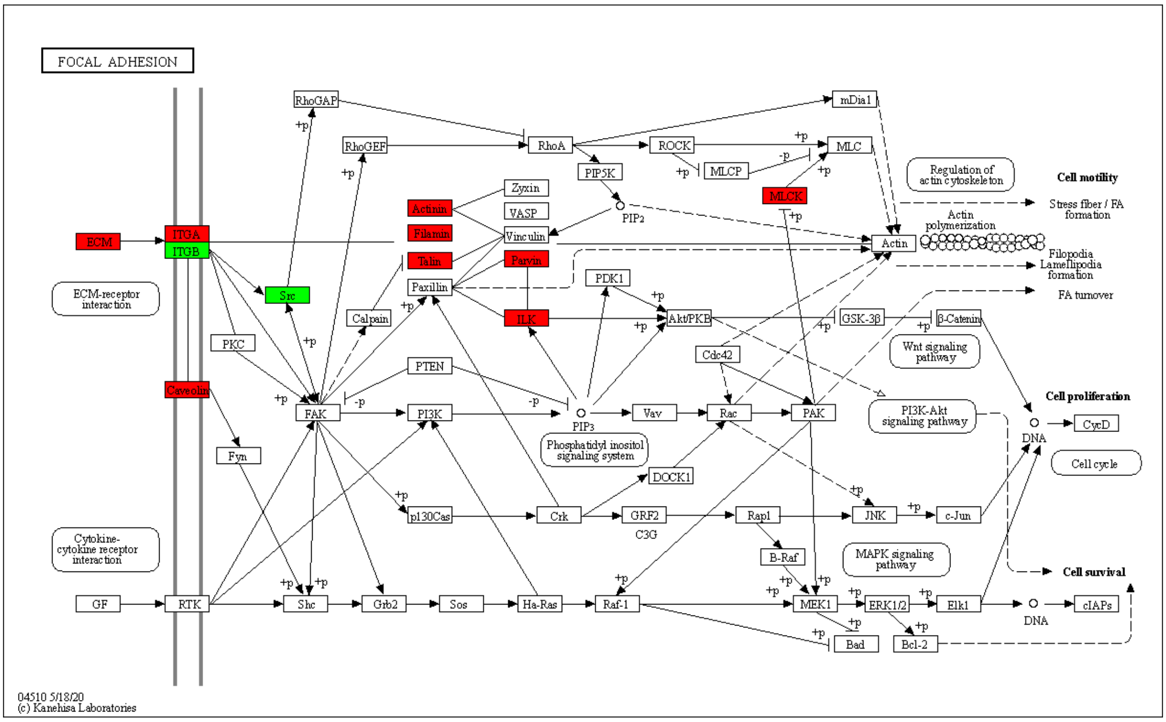

Figure S2: Graphical network map of KEGG *Focal adhesion* pathway. Red, up-regulated proteins; green, down-regulated proteins.

Raw file	Scan number	Mass analyzer	Score	m/z	Proteins
B1453260_Slot2−16_1_10790	50830	TOF	178.65	827.402	Q9BZQ8

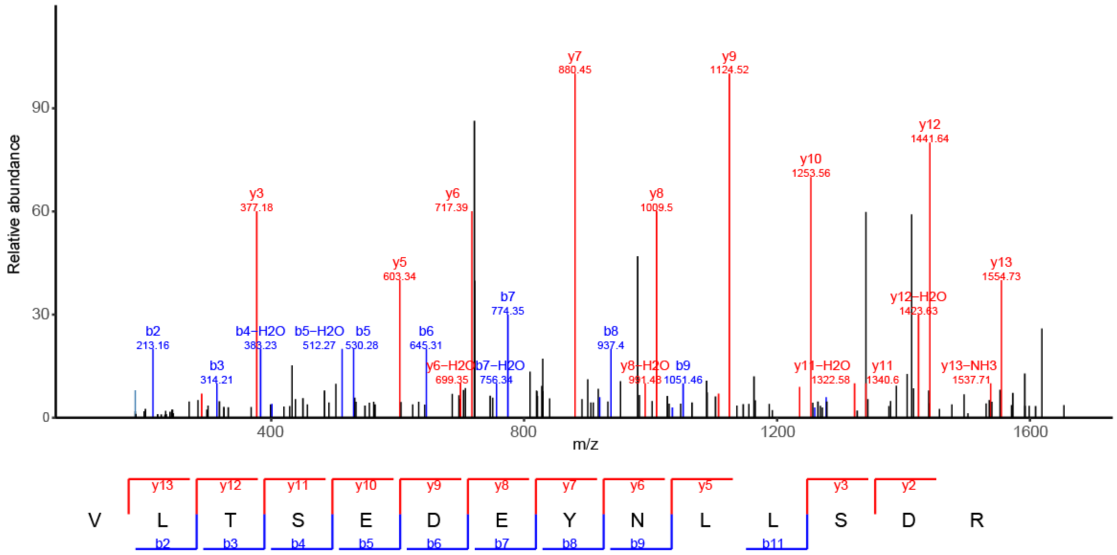

Figure S3: The mass spectrum of NIBAN1 unique peptide (VLTSEDEYNLLSDR).

Table S1: The clinicopathological information associated with paired primary/recurrent specimens

Case. No	Sex	Age	Time. diagnosis	Time. recurrence	Time to recurrence （months）	Tumor stage
BC018	M	47	2018.1	2020.3	26	Ta
BC033	M	30	2018.5	2019.4	11	T1
BC893	M	53	2018.7	2019.3	8	T1
BC260	M	35	2019.1	2020.6	18	T1
BC258	F	68	2019.9	2020.6	9	T1
BC764	F	69	2019.11	2021.4	17	T1
BC393	M	77	2020.4	2020.11	7	T1
BC097	M	69	2017.12	2019.11	23	T1

Table S2: Differentially expressed proteins in recurrent vs. primary comparisons

Protein accession	Gene name	R/P Ratio	Log2 R/P	R/P P value	Regulated Type
Q9BZQ8	NIBAN1	7.33	2.87	0.008	Up
O15061	SYNM	6.57	2.72	0.023	Up
P08648	ITGA5	6.20	2.63	0.008	Up
P00746	CFD	4.71	2.24	0.015	Up
Q15124	PGM5	4.69	2.23	0.025	Up
P30837	ALDH1B1	4.40	2.14	0.030	Up
Q9HBL0	TNS1	3.96	1.98	0.023	Up
P55083	MFAP4	3.92	1.97	0.028	Up
P46821	MAP1B	3.83	1.94	0.020	Up
P02511	CRYAB	3.74	1.90	0.009	Up
Q13642	FHL1	3.57	1.84	0.045	Up
P63267	ACTG2	3.55	1.83	0.035	Up
P21291	CSRP1	3.55	1.83	0.013	Up
P35749	MYH11	3.55	1.83	0.041	Up
Q9NR12	PDLIM7	3.54	1.82	0.031	Up
Q86VB7	CD163	3.45	1.79	0.021	Up
Q8IVN8	SBSPON	3.40	1.77	0.035	Up
P62736	ACTA2	3.34	1.74	0.040	Up
P22352	GPX3	3.21	1.68	0.009	Up
.0A0A0MRZ	IGKV3D-11	3.20	1.68	0.037	Up
Q14195	DPYSL3	3.05	1.61	0.004	Up
Q96PD5	PGLYRP2	2.99	1.58	0.034	Up
Q9NZU5	LMCD1	2.99	1.58	0.008	Up
P01861	IGHG4	2.94	1.56	0.027	Up
P00748	F12	2.92	1.55	0.034	Up
O43866	CD5L	2.92	1.55	0.010	Up
P35858	IGFALS	2.86	1.52	0.029	Up
Q14BN4	SLMAP	2.85	1.51	0.019	Up
P21810	BGN	2.78	1.48	0.038	Up
Q15746	MYLK	2.71	1.44	0.017	Up
P12110	COL6A2	2.71	1.44	0.019	Up
P0DOX7	--	2.64	1.40	0.038	Up
P12109	COL6A1	2.63	1.40	0.019	Up
P21333	FLNA	2.63	1.40	0.040	Up
Q8TF30	WHAMM	2.62	1.39	0.035	Up
P01860	IGHG3	2.58	1.37	0.040	Up
P01859	IGHG2	2.57	1.36	0.026	Up
P01009	SERPINA1	2.57	1.36	0.007	Up
P02753	RBP4	2.54	1.35	0.017	Up
P02774	GC	2.52	1.33	0.004	Up
P10301	RRAS	2.51	1.33	0.021	Up
Q63ZY3	KANK2	2.51	1.33	0.031	Up
Q96AC1	FERMT2	2.47	1.30	0.013	Up
P01019	AGT	2.46	1.30	0.020	Up
O43182	ARHGAP6	2.46	1.30	0.020	Up
Q8WX93	PALLD	2.44	1.29	0.049	Up
O95810	CAVIN2	2.44	1.29	0.024	Up
Q96P44	COL21A1	2.35	1.23	0.048	Up

P0DOX8	--	2.35	1.23	0.047	Up
Q13418	ILK	2.32	1.22	0.015	Up
P53814	SMTN	2.32	1.21	0.005	Up
Q9NZN4	EHD2	2.30	1.20	0.033	Up
P27144	AK4	2.28	1.19	0.012	Up
Q03135	CAV1	2.26	1.18	0.008	Up
P00488	F13A1	2.26	1.18	0.005	Up
P01023	A2M	2.25	1.17	0.046	Up
P12111	COL6A3	2.23	1.16	0.029	Up
P27169	PON1	2.22	1.15	0.015	Up
P08133	ANXA6	2.22	1.15	0.021	Up
Q9Y6C2	EMILIN1	2.18	1.12	0.013	Up
P35555	FBN1	2.17	1.12	0.021	Up
O75923	DYSF	2.16	1.11	0.011	Up
P02787	TF	2.14	1.10	0.015	Up
P04196	HRG	2.14	1.10	0.032	Up
P02790	HPX	2.07	1.05	0.013	Up
P43121	MCAM	2.04	1.03	0.049	Up
P02765	AHSG	2.03	1.02	0.020	Up
Q14061	COX17	2.01	1.01	0.030	Up
P50479	PDLIM4	1.99	0.99	0.005	Up
P09382	LGALS1	1.98	0.99	0.009	Up
Q16363	LAMA4	1.98	0.98	0.046	Up
Q9NVD7	PARVA	1.97	0.98	0.032	Up
P55001	MFAP2	1.94	0.95	0.039	Up
P22897	MRC1	1.92	0.94	0.049	Up
P60033	CD81	1.91	0.94	0.033	Up
O95425	SVIL	1.84	0.88	0.028	Up
P48059	LIMS1	1.81	0.86	0.047	Up
Q15555	MAPRE2	1.81	0.86	0.011	Up
P02647	APOA1	1.81	0.85	0.040	Up
P01008	SERPINC1	1.78	0.83	0.048	Up
P04350	TUBB4A	1.76	0.81	0.039	Up
Q9ULV4	CORO1C	1.75	0.81	0.039	Up
Q9BUF5	TUBB6	1.75	0.80	0.029	Up
Q9Y490	TLN1	1.73	0.79	0.022	Up
O95832	CLDN1	1.73	0.79	0.030	Up
Q13228	SELENBP1	1.72	0.79	0.024	Up
Q07954	LRP1	1.72	0.78	0.046	Up
Q93052	LPP	1.70	0.77	0.005	Up
P12814	ACTN1	1.69	0.76	0.023	Up
Q16881	TXNRD1	1.69	0.76	0.023	Up
P21980	TGM2	1.68	0.75	0.028	Up
Q9Y305	ACOT9	1.68	0.75	0.039	Up
Q92615	LARP4B	1.68	0.74	0.034	Up
P08670	VIM	1.67	0.74	0.041	Up
P08758	ANXA5	1.66	0.73	0.012	Up
P26038	MSN	1.65	0.72	0.003	Up
P60660	MYL6	1.65	0.72	0.010	Up
Q16555	DPYSL2	1.63	0.70	0.041	Up
P42785	PRCP	1.62	0.70	0.027	Up

Q9Y696	CLIC4	1.62	0.69	0.031	Up
P51452	DUSP3	1.62	0.69	0.006	Up
Q96LD4	TRIM47	1.60	0.68	0.009	Up
P61601	NCALD	1.59	0.67	0.003	Up
Q13425	SNTB2	1.54	0.62	0.003	Up
Q9Y5X1	SNX9	1.53	0.61	0.007	Up
P40855	PEX19	0.67	-0.59	0.029	Down
Q9BVL2	NUP58	0.67	-0.59	0.030	Down
Q9GZZ1	NAA50	0.67	-0.59	0.044	Down
P23588	EIF4B	0.67	-0.59	0.012	Down
O15305	PMM2	0.66	-0.59	0.048	Down
Q6PKG0	LARP1	0.66	-0.60	0.012	Down
O60832	DKC1	0.66	-0.60	0.035	Down
Q9H0D6	XRN2	0.66	-0.60	0.032	Down
P12931	SRC	0.66	-0.60	0.036	Down
P42167	TMPO	0.66	-0.60	0.043	Down
O75844	ZMPSTE24	0.66	-0.60	0.033	Down
Q9Y606	PUS1	0.66	-0.60	0.015	Down
P49736	MCM2	0.66	-0.60	0.027	Down
Q9GZM7	TINAGL1	0.66	-0.61	0.009	Down
Q12769	NUP160	0.66	-0.61	0.006	Down
Q12929	EPS8	0.66	-0.61	0.005	Down
O94776	MTA2	0.66	-0.61	0.007	Down
Q9BYD1	MRPL13	0.66	-0.61	0.005	Down
Q53GS9	USP39	0.65	-0.61	0.025	Down
P52948	NUP98	0.65	-0.61	0.017	Down
O75494	SRSF10	0.65	-0.62	0.003	Down
Q14134	TRIM29	0.65	-0.62	0.020	Down
P98175	RBM10	0.65	-0.62	0.003	Down
Q07812	BAX	0.65	-0.62	0.012	Down
P55957	BID	0.65	-0.62	0.013	Down
Q8WVV4	POF1B	0.65	-0.62	0.031	Down
Q9BWF3	RBM4	0.65	-0.63	0.017	Down
P57740	NUP107	0.65	-0.63	0.003	Down
Q9HAV4	XPO5	0.65	-0.63	0.014	Down
Q9HAW9	UGT1A8	0.65	-0.63	0.024	Down
Q8N3C0	ASCC3	0.65	-0.63	0.014	Down
Q9NW64	RBM22	0.65	-0.63	0.017	Down
P30519	HMOX2	0.65	-0.63	0.046	Down
Q96HE7	ERO1A	0.65	-0.63	0.003	Down
Q15050	RRS1	0.64	-0.63	0.035	Down
P11117	ACP2	0.64	-0.64	0.000	Down
P78347	GTF2I	0.64	-0.64	0.037	Down
Q15427	SF3B4	0.64	-0.64	0.038	Down
Q08174	PCDH1	0.64	-0.64	0.012	Down
Q9NZ43	USE1	0.64	-0.64	0.035	Down
O95994	AGR2	0.64	-0.64	0.017	Down
Q6UN15	FIP1L1	0.64	-0.65	0.010	Down
Q7L5L3	GDPD3	0.64	-0.65	0.042	Down
Q12931	TRAP1	0.64	-0.65	0.002	Down
Q9BQ67	GRWD1	0.64	-0.65	0.021	Down

Q14157	UBAP2L	0.63	-0.66	0.020	Down
Q9NR30	DDX21	0.63	-0.66	0.012	Down
Q14694	USP10	0.63	-0.66	0.028	Down
Q6P1J9	CDC73	0.63	-0.67	0.007	Down
Q9BRG1	VPS25	0.63	-0.67	0.036	Down
Q10570	CPSF1	0.63	-0.67	0.041	Down
Q9Y5B9	SUPT16H	0.63	-0.67	0.021	Down
Q9Y2W2	WBP11	0.62	-0.68	0.018	Down
P09497	CLTB	0.62	-0.69	0.028	Down
P35269	GTF2F1	0.62	-0.70	0.004	Down
P34897	SHMT2	0.61	-0.70	0.047	Down
Q05519	SRSF11	0.61	-0.70	0.035	Down
Q8IZL8	PELP1	0.61	-0.71	0.009	Down
Q8TEM1	NUP210	0.61	-0.71	0.017	Down
Q99729	HNRNPAB	0.61	-0.71	0.002	Down
Q9ULT8	HECTD1	0.61	-0.71	0.010	Down
Q96SB4	SRPK1	0.60	-0.73	0.042	Down
Q12972	PPP1R8	0.60	-0.73	0.003	Down
Q8ND30	PPFIBP2	0.60	-0.73	0.045	Down
O14976	GAK	0.60	-0.74	0.005	Down
P60468	SEC61B	0.60	-0.74	0.001	Down
Q96E39	RBMXL1	0.60	-0.74	0.049	Down
Q14137	BOP1	0.60	-0.74	0.012	Down
Q14728	MFSD10	0.60	-0.74	0.001	Down
O15269	SPTLC1	0.60	-0.74	0.028	Down
P33993	MCM7	0.59	-0.76	0.043	Down
Q9P258	RCC2	0.59	-0.77	0.010	Down
Q9NWT6	HIF1AN	0.59	-0.77	0.038	Down
P10586	PTPRF	0.58	-0.77	0.025	Down
P18440	NAT1	0.58	-0.77	0.019	Down
Q5JTH9	RRP12	0.58	-0.78	0.012	Down
P18754	RCC1	0.58	-0.79	0.008	Down
Q8N573	OXR1	0.58	-0.80	0.022	Down
Q8TEX9	IPO4	0.58	-0.80	0.002	Down
P18564	ITGB6	0.57	-0.80	0.007	Down
Q03169	TNFAIP2	0.57	-0.81	0.024	Down
Q8NFF5	FLAD1	0.57	-0.82	0.029	Down
Q9Y3T9	NOC2L	0.57	-0.82	0.004	Down
Q86YP4	GATAD2A	0.57	-0.82	0.006	Down
Q6ZXV5	TMTC3	0.57	-0.82	0.000	Down
P25205	MCM3	0.56	-0.83	0.041	Down
Q9UHN6	CEMIP2	0.56	-0.83	0.026	Down
P49915	GMPS	0.56	-0.84	0.017	Down
P39748	FEN1	0.56	-0.84	0.043	Down
Q13895	BYSL	0.56	-0.84	0.003	Down
Q04864	REL	0.56	-0.85	0.017	Down
Q13315	ATM	0.55	-0.85	0.033	Down
Q9Y673	ALG5	0.55	-0.86	0.027	Down
Q96CM8	ACSF2	0.54	-0.88	0.029	Down
P62306	SNRPF	0.54	-0.89	0.005	Down
Q4G176	ACSF3	0.54	-0.90	0.006	Down

Q96QV6	H2AC1	0.53	-0.91	0.011	Down
P12004	PCNA	0.53	-0.91	0.031	Down
Q9H3D4	TP63	0.53	-0.92	0.017	Down
Q8WY22	BRI3BP	0.53	-0.93	0.014	Down
Q9Y2L1	DIS3	0.51	-0.96	0.014	Down
O15347	HMGB3	0.51	-0.99	0.034	Down
Q8NF37	LPCAT1	0.50	-0.99	0.016	Down
Q8N142	ADSS1	0.49	-1.02	0.041	Down
P03956	MMP1	0.49	-1.02	0.045	Down
P32519	ELF1	0.49	-1.03	0.031	Down
Q2NL82	TSR1	0.48	-1.05	0.015	Down
Q9UPQ0	LIMCH1	0.48	-1.06	0.021	Down
Q9P2M7	CGN	0.45	-1.14	0.026	Down
Q13751	LAMB3	0.42	-1.26	0.013	Down
P02786	TFRC	0.42	-1.26	0.016	Down
Q14978	NOLC1	0.41	-1.29	0.012	Down
P68431	H3C1	0.33	-1.62	0.044	Down

Table S3: Differentially expressed genes in shNIBAN1 vs. Scr comparison

AccID	Fold change	log2 (Fold change)	P value
FAM192BP	0.060	-4.07	0.015
RPL35AP21	0.087	-3.52	0.030
HSP90AA4P	0.110	-3.19	0.001
EQTN	0.111	-3.18	0.010
WNT7A	0.113	-3.15	0.001
TPRKBP2	0.115	-3.12	0.020
RNU4-50P	0.116	-3.10	0.002
CNR1	0.121	-3.05	0.016
PROX1	0.121	-3.04	0.006
CSF2RB	0.124	-3.01	0.004
RFESDP1	0.145	-2.79	0.015
OXCT2	0.151	-2.73	0.050
TMC5	0.155	-2.69	0.009
MNX1-AS2	0.158	-2.66	0.017
ILK	0.160	-2.64	0.000
TPSG1	0.165	-2.60	0.037
SRRM2-AS1	0.183	-2.45	0.014
FAM24B	0.189	-2.40	0.004
GALNTL6	0.193	-2.38	0.018
FP565260.[illegible]	0.194	-2.37	0.006
MEDAG	0.202	-2.30	0.016
CRLF1	0.203	-2.30	0.010
NIBAN1	0.204	-2.29	0.000
ABCC8	0.207	-2.27	0.009
ACTG1P15	0.209	-2.26	0.044
TSSC2	0.211	-2.24	0.040
DLL3	0.226	-2.15	0.048
EDN2	0.231	-2.11	0.004
POLRMTP1	0.240	-2.06	0.013
NOG	0.250	-2.00	0.002
HSD17B2	0.250	-2.00	0.006
C6orf118	0.251	-1.99	0.028
DUSP9	0.265	-1.92	0.014
C21orf59	0.265	-1.92	0.025
CDH3	0.265	-1.92	0.001
SYN3	0.265	-1.92	0.018
WNT7B	0.265	-1.91	0.004
SEMA7A	0.267	-1.91	0.001
SEMA5A-AS[illegible]	0.267	-1.90	0.015
ACE2	0.274	-1.87	0.041
ROPN1L	0.277	-1.85	0.015
CCNB1	0.278	-1.85	0.000
TRPM6	0.279	-1.84	0.033
CCDC107	0.281	-1.83	0.028
CDH1	0.288	-1.80	0.009
CRYGN	0.288	-1.79	0.023
FMO3	0.291	-1.78	0.041
GUSBP5	0.291	-1.78	0.040
FTH1P15	0.294	-1.77	0.040
E2F8	0.295	-1.76	0.013
MYL6	0.297	-1.75	0.002
CCND1	0.297	-1.75	0.000

DDN	0.300	-1.74	0.000
PDGFRB	0.300	-1.74	0.003
STX1B	0.303	-1.72	0.034
SLC2A5	0.308	-1.70	0.044
PA2G4P2	0.308	-1.70	0.037
CAV1	0.311	-1.69	0.000
ADAMTS15	0.315	-1.67	0.006
PHOSPHO1	0.324	-1.62	0.021
ITGA3	0.327	-1.61	0.000
ST13P2	0.328	-1.61	0.032
ITGA6	0.330	-1.60	0.001
CT62	0.330	-1.60	0.031
RND1	0.331	-1.60	0.040
PPL	0.334	-1.58	0.005
RPS20P22	0.335	-1.58	0.026
COL4A1	0.335	-1.58	0.000
EGLN3	0.335	-1.58	0.000
FAM46B	0.339	-1.56	0.005
CXCR4	0.341	-1.55	0.034
RPS27AP13	0.342	-1.55	0.037
MKI67	0.342	-1.55	0.000
H19	0.345	-1.54	0.001
CPA4	0.345	-1.53	0.012
IIRLET7BH(	0.346	-1.53	0.036
STAG3L5P	0.346	-1.53	0.033
BCL2	0.346	-1.53	0.001
GPLD1	0.347	-1.53	0.005
SMIM1	0.354	-1.50	0.042
SOCS1	0.354	-1.50	0.031
C7orf69	0.359	-1.48	0.002
ERBB3	0.359	-1.48	0.000
MGARP	0.361	-1.47	0.001
KRT8P3	0.363	-1.46	0.009
WISP2	0.366	-1.45	0.032
KCNJ8	0.366	-1.45	0.029
PLEKHS1	0.366	-1.45	0.014
VASH2	0.370	-1.43	0.006
GJA5	0.370	-1.43	0.001
PTPRQ	0.371	-1.43	0.025
HILPDA	0.372	-1.43	0.000
RRAD	0.374	-1.42	0.027
SOWAHD	0.376	-1.41	0.012
PCDH10	0.376	-1.41	0.025
OLFML1	0.377	-1.41	0.002
CYP2E1	0.381	-1.39	0.041
LFNG	0.382	-1.39	0.012
ZG16B	0.382	-1.39	0.048
ACTN1	0.387	-1.37	0.004
PKD1L1	0.390	-1.36	0.037
LAMA4	0.391	-1.36	0.002
FN1	0.391	-1.36	0.001
LINP1	0.393	-1.35	0.015
C10orf10	0.396	-1.34	0.034
NPPB	0.397	-1.33	0.008

ASB9	0.398	-1.33	0.029
RAB26	0.398	-1.33	0.012
PDGFB	0.398	-1.33	0.003
CLIC3	0.401	-1.32	0.007
CACNG7	0.408	-1.29	0.044
KRT8P10	0.408	-1.29	0.050
THBS1	0.409	-1.29	0.000
SNORA7	0.412	-1.28	0.033
SNORA7	0.412	-1.28	0.033
SNORA7	0.412	-1.28	0.033
SNORA7	0.412	-1.28	0.033
SNORA7	0.412	-1.28	0.033
ARNT2	0.412	-1.28	0.003
APLN	0.412	-1.28	0.019
KIF20A	0.416	-1.27	0.003
PPFIA4	0.416	-1.26	0.011
KIAA1755	0.419	-1.26	0.001
PNPLA3	0.419	-1.25	0.012
WT1	0.420	-1.25	0.027
HSD3B1	0.426	-1.23	0.000
OR2I1P	0.435	-1.20	0.015
C7orf61	0.438	-1.19	0.017
PFKFB3	0.441	-1.18	0.001
LBH	0.445	-1.17	0.000
DDIT4L	0.446	-1.17	0.007
TIMP3	0.446	-1.17	0.002
LZTS1	0.450	-1.15	0.026
CD24	0.453	-1.14	0.000
PGF	0.455	-1.13	0.039
PDK1	0.456	-1.13	0.006
SLC29A4	0.459	-1.12	0.049
BMP4	0.460	-1.12	0.024
MGAM	0.462	-1.12	0.000
ARHGAP28	0.464	-1.11	0.002
HSP90AA2P	0.465	-1.11	0.026
CGN	0.466	-1.10	0.004
VCAM1	0.466	-1.10	0.002
SLC4A11	0.466	-1.10	0.005
SHROOM3	0.467	-1.10	0.031
SUCNR1	0.468	-1.10	0.001
PKP2	0.469	-1.09	0.009
FOS	0.469	-1.09	0.030
C1QL4	0.471	-1.09	0.018
PARD6A	0.472	-1.08	0.008
RASD2	0.475	-1.07	0.024
HK2	0.475	-1.07	0.001
WIPF3	0.477	-1.07	0.015
KDM4A-AS1	0.477	-1.07	0.018
TWIST1	0.478	-1.06	0.037
COL3A1	0.479	-1.06	0.000
SCO2	0.479	-1.06	0.005
SCO2	0.479	-1.06	0.005
THAP8	0.480	-1.06	0.001
PMF1	0.483	-1.05	0.022

ZNF488	0.484	-1.05	0.021
CDKN1C	0.486	-1.04	0.047
POU3F2	0.489	-1.03	0.020
NDRG1	0.490	-1.03	0.000
DAB2	0.490	-1.03	0.001
RGMB-AS1	0.491	-1.03	0.020
CGNL1	0.493	-1.02	0.007
NACAD	0.494	-1.02	0.008
CCDC7	0.495	-1.02	0.014
KRT18	0.495	-1.01	0.002
SLCO2A1	0.496	-1.01	0.000
MAP1A	0.497	-1.01	0.010
ATP7B	0.498	-1.01	0.001
GOLGA6L10	2.001	1.00	0.023
NRCAM	2.002	1.00	0.004
SNHG7	2.003	1.00	0.013
HOXA5	2.007	1.01	0.046
GEM	2.011	1.01	0.037
CAVIN4	2.011	1.01	0.015
RGPD3	2.012	1.01	0.009
TXNL4B	2.027	1.02	0.002
TRIM66	2.027	1.02	0.003
SPDYE5	2.027	1.02	0.019
IFI44L	2.028	1.02	0.000
LAMP3	2.030	1.02	0.003
PHLDA1	2.035	1.02	0.000
C16orf71	2.042	1.03	0.017
TMEM154	2.045	1.03	0.024
CLGN	2.050	1.04	0.003
CASC9	2.056	1.04	0.018
GABPB1-AS1	2.060	1.04	0.004
SPTA1	2.061	1.04	0.011
CPEB1-AS1	2.064	1.05	0.017
NBPF12	2.065	1.05	0.008
RCBTB1	2.069	1.05	0.000
POU6F1	2.069	1.05	0.028
RP9P	2.070	1.05	0.036
FLJ37453	2.074	1.05	0.017
MSC-AS1	2.077	1.05	0.003
IL6	2.079	1.06	0.000
KIAA1217	2.083	1.06	0.005
HDAC9	2.088	1.06	0.023
RCBTB2	2.088	1.06	0.014
GLI1	2.095	1.07	0.009
CHAC1	2.096	1.07	0.012
TXNIP	2.098	1.07	0.002
LMOD3	2.108	1.08	0.011
IFITM10	2.108	1.08	0.012
ACY3	2.108	1.08	0.023
RSPO3	2.110	1.08	0.039
PXYLP1	2.113	1.08	0.000
CDKL3	2.126	1.09	0.041
SNHG20	2.130	1.09	0.013
HOXB-AS4	2.130	1.09	0.018

TMEM199	2.133	1.09	0.004
DNHD1	2.134	1.09	0.002
IL1B	2.141	1.10	0.008
EEF1AKMT1	2.147	1.10	0.008
HOXC13-AS	2.149	1.10	0.006
INTS6-AS1	2.154	1.11	0.005
CACNA2D2	2.154	1.11	0.043
FUT1	2.161	1.11	0.025
SCAMP1-AS	2.165	1.11	0.004
SOCS2	2.167	1.12	0.030
RNASEK	2.168	1.12	0.019
HIST1H2BD	2.172	1.12	0.002
C8orf76	2.173	1.12	0.012
CHRNA10	2.178	1.12	0.000
RND3	2.179	1.12	0.000
CA5B	2.184	1.13	0.017
TTLL6	2.195	1.13	0.047
GOLGA8N	2.211	1.14	0.029
SLCO4C1	2.228	1.16	0.002
BNC1	2.234	1.16	0.006
SAT1	2.244	1.17	0.000
MAP2	2.246	1.17	0.004
TSSK5P	2.246	1.17	0.000
KRCC1	2.254	1.17	0.004
YBEY	2.257	1.17	0.011
CXCL5	2.263	1.18	0.003
ZNF793-AS	2.265	1.18	0.041
LTB4R	2.268	1.18	0.006
NMRAL2P	2.272	1.18	0.002
ADM2	2.276	1.19	0.005
ZNF594	2.278	1.19	0.000
GUCY2C	2.286	1.19	0.022
QRICH2	2.308	1.21	0.009
ICA1	2.309	1.21	0.049
CXCL8	2.312	1.21	0.001
PRELID3A	2.328	1.22	0.026
BDKRB1	2.330	1.22	0.007
WDR78	2.331	1.22	0.012
NECTIN4	2.338	1.23	0.006
SERPINB9P	2.339	1.23	0.002
BEST1	2.343	1.23	0.008
HIST1H3H	2.345	1.23	0.014
ARHGEF34P	2.360	1.24	0.008
OR7E14P	2.370	1.24	0.012
HIST1H2AC	2.376	1.25	0.000
IFIT2	2.383	1.25	0.001
RPL29P19	2.395	1.26	0.002
MYPN	2.397	1.26	0.002
BNIP3P17	2.401	1.26	0.021
SLFN5	2.408	1.27	0.015
CSGALNACT	2.434	1.28	0.039
CD22	2.440	1.29	0.006
CFAP57	2.441	1.29	0.050
KYNU	2.463	1.30	0.016

GABRR3	2.465	1.30	0.003
LEPR	2.479	1.31	0.001
CCL3L3	2.486	1.31	0.023
TRIB3	2.488	1.31	0.001
U62317.1	2.492	1.32	0.004
GPR35	2.498	1.32	0.020
FRZB	2.519	1.33	0.003
CARD14	2.519	1.33	0.016
POLN	2.548	1.35	0.030
CYP1B1-AS1	2.576	1.36	0.002
RAET1G	2.585	1.37	0.030
CACNB2	2.586	1.37	0.047
MAP3K7CL	2.591	1.37	0.049
NPIPA5	2.593	1.37	0.014
B3GNT5	2.596	1.38	0.013
HIST1H2BH	2.599	1.38	0.026
FBXO39	2.614	1.39	0.046
GNMT	2.626	1.39	0.006
TNXB	2.642	1.40	0.026
C1orf229	2.642	1.40	0.024
GLP2R	2.642	1.40	0.017
NAALADL2	2.654	1.41	0.026
HYPK	2.654	1.41	0.012
RIBC1	2.661	1.41	0.033
SMTNL1	2.674	1.42	0.007
DUSP2	2.677	1.42	0.001
VEGFD	2.680	1.42	0.015
NUS1P1	2.690	1.43	0.010
NUP50-AS1	2.691	1.43	0.004
OR10AB1P	2.702	1.43	0.020
RSAD2	2.724	1.45	0.001
FAM229A	2.729	1.45	0.000
KLHDC7B	2.735	1.45	0.000
CXCL3	2.763	1.47	0.014
ADTRP	2.780	1.48	0.032
DENND6B	2.800	1.49	0.007
LY6G5B	2.804	1.49	0.046
RAB40A	2.819	1.50	0.003
HIST1H4H	2.824	1.50	0.007
FAM86FP	2.841	1.51	0.014
VSIG8	2.855	1.51	0.043
LPL	2.861	1.52	0.007
PATL2	2.888	1.53	0.013
DHRS2	2.903	1.54	0.004
MAGI2	2.907	1.54	0.012
NR4A3	2.922	1.55	0.015
LSMEM1	2.923	1.55	0.018
CEACAMP10	2.945	1.56	0.001
CUZD1	2.955	1.56	0.001
NBPF25P	2.963	1.57	0.043
HIST2H2BF	2.980	1.58	0.046
SDCBP2	2.998	1.58	0.021
SMOX	3.006	1.59	0.003
CXCL2	3.015	1.59	0.000

IL24	3.016	1.59	0.000
BAX	3.035	1.60	0.045
NEURL3	3.066	1.62	0.024
KLK14	3.077	1.62	0.046
HNRNPA1P3[illegible]	3.105	1.63	0.022
ATP2A1	3.130	1.65	0.037
CFAP43	3.166	1.66	0.036
[illegible]LGAP1-AS[illegible]	3.169	1.66	0.001
P2RX7	3.172	1.67	0.009
ITGB1-DT	3.221	1.69	0.010
MIR25	3.231	1.69	0.028
IFITM4P	3.271	1.71	0.009
[illegible]CVR2B-AS[illegible]	3.276	1.71	0.024
PTGS2	3.295	1.72	0.000
HIST1H2BE	3.309	1.73	0.035
CSF3	3.333	1.74	0.015
RBKS	3.346	1.74	0.004
CCDC88B	3.347	1.74	0.046
RNF39	3.351	1.74	0.033
USP6	3.357	1.75	0.030
DNAH17	3.369	1.75	0.018
LTB4R2	3.373	1.75	0.029
CD80	3.383	1.76	0.031
ZBED3-AS1	3.386	1.76	0.002
HIST1H4K	3.403	1.77	0.045
LY96	3.410	1.77	0.002
EPHA10	3.449	1.79	0.016
ZNF425	3.468	1.79	0.006
INHBE	3.491	1.80	0.014
PIWIL4	3.542	1.82	0.000
NUPR1	3.544	1.83	0.015
C2	3.546	1.83	0.042
COL4A3	3.618	1.86	0.019
USP30-AS1	3.635	1.86	0.005
PDE7B	3.647	1.87	0.036
CNTF	3.703	1.89	0.040
[illegible]NAJC9-AS[illegible]	3.703	1.89	0.023
[illegible]NAJC9-AS[illegible]	3.703	1.89	0.023
VIPR1	3.760	1.91	0.033
IL33	3.812	1.93	0.000
GDF15	3.842	1.94	0.000
ORF4L2-AS	3.842	1.94	0.046
SLC11A1	3.875	1.95	0.044
SPART-AS1	3.890	1.96	0.015
ADGRF5P1	3.914	1.97	0.025
SLC7A7	3.930	1.97	0.015
CFAP53	3.953	1.98	0.015
RAMP2-AS1	4.017	2.01	0.002
KLRD1	4.024	2.01	0.027
MCPH1-AS1	4.041	2.01	0.043
AK8	4.070	2.02	0.020
CPHL1P	4.078	2.03	0.013
ADGRF4	4.080	2.03	0.008
DRP2	4.092	2.03	0.009

EFCAB8	4.119	2.04	0.009
PJVK	4.142	2.05	0.032
CFAP52	4.194	2.07	0.022
KIAA1683	4.218	2.08	0.010
BEST3	4.227	2.08	0.010
APBA1	4.239	2.08	0.041
CCL4L2	4.239	2.08	0.028
OVGP1	4.242	2.08	0.020
C9orf139	4.242	2.08	0.018
PLIN1	4.303	2.11	0.008
PID1	4.400	2.14	0.004
TMEM220	4.412	2.14	0.042
CALB1	4.433	2.15	0.007
LEKR1	4.435	2.15	0.037
SPATA9	4.436	2.15	0.025
THEM5	4.554	2.19	0.049
MTG1	4.735	2.24	0.011
ATP2B1-AS1	4.782	2.26	0.006
ILDR2	4.808	2.27	0.033
CLDN9	4.839	2.27	0.010
VILL	4.923	2.30	0.018
COLQ	4.974	2.31	0.021
KIZ-AS1	5.036	2.33	0.012
LVRN	5.077	2.34	0.010
PGM5	5.087	2.35	0.045
INTS4P2	5.130	2.36	0.018
HIST1H3J	5.156	2.37	0.029
GRM1	5.197	2.38	0.041
IFNA20P	5.203	2.38	0.045
MEM254-AS	5.232	2.39	0.004
GHRLOS	5.284	2.40	0.038
GHRLOS	5.284	2.40	0.038
SSPO	5.297	2.41	0.008
SNORD63	5.310	2.41	0.004
SNORD63	5.310	2.41	0.004
SNORD63	5.310	2.41	0.004
TRHR	5.391	2.43	0.027
CXADRP3	5.435	2.44	0.004
LRRC74B	5.510	2.46	0.025
BSN-AS2	5.572	2.48	0.007
MMP3	5.689	2.51	0.002
BAD	5.821	2.54	0.040
PROX2	5.830	2.54	0.041
OLAH	5.832	2.54	0.022
ZFP2	5.845	2.55	0.025
FAM69B	5.868	2.55	0.004
RTL9	5.985	2.58	0.009
IL11	5.994	2.58	0.000
MIR196A1	6.089	2.61	0.018
OXCT1-AS1	6.124	2.61	0.002
NR2E3	6.193	2.63	0.017
SVILP1	6.442	2.69	0.038
RBMS3-AS3	6.541	2.71	0.028
PSMG3-AS1	6.715	2.75	0.010

DKK2	6.761	2.76	0.012
IMPG2	6.842	2.77	0.035
HPX	7.006	2.81	0.014
CARS-AS1	7.136	2.84	0.021
EPS8L1	7.225	2.85	0.008
BLOC1S1	7.261	2.86	0.022
SLC9B1	7.405	2.89	0.028
TOLLIP-AS	7.490	2.90	0.000
LHB	7.518	2.91	0.045
DRAXIN	7.526	2.91	0.021
MEIOB	7.581	2.92	0.045
ZNF705E	7.608	2.93	0.011
CD37	7.844	2.97	0.001
PLXDC1	7.850	2.97	0.017
GRIN2C	8.013	3.00	0.040
FOXD4L1	8.284	3.05	0.012
IL1RN	8.324	3.06	0.002
PPP1R14D	8.412	3.07	0.017
ARRDC3-AS	8.622	3.11	0.013
MKRN7P	8.657	3.11	0.019
WDR97	8.695	3.12	0.004
GPR61	8.700	3.12	0.010
ABCA10	8.788	3.14	0.006
IL36G	8.889	3.15	0.014
CDRT15	8.899	3.15	0.014
PLSCR2	9.056	3.18	0.004
MAFA	9.060	3.18	0.009
SBSN	9.362	3.23	0.004
KRTAP5-1	9.368	3.23	0.032
DMGDH	9.487	3.25	0.003
GRM2	9.573	3.26	0.033
RN7SKP70	9.594	3.26	0.003
CCDC116	9.754	3.29	0.026
DISP3	10.067	3.33	0.003
CD44-AS1	10.613	3.41	0.004
KRTAP21-2	10.846	3.44	0.004
PAX5	11.189	3.48	0.009
MIR4482	11.506	3.52	0.002
PLA2G10	11.702	3.55	0.026
CFP	12.678	3.66	0.038
BDNF-AS	13.790	3.79	0.038
CPNE9	14.801	3.89	0.023
DDX10P1	14.931	3.90	0.001
BTK	15.229	3.93	0.020
GALNT12	15.476	3.95	0.012
TEX52	15.507	3.95	0.015
HCG9P5	16.031	4.00	0.027
MYO1H	16.397	4.04	0.003
AXDND1	17.516	4.13	0.044
HM13-AS1	17.857	4.16	0.048
BSN	18.268	4.19	0.049
IFNB1	19.125	4.26	0.033
PXDNL	19.424	4.28	0.036
AKAP3	19.574	4.29	0.039

HIST1H2BG	20.290	4.34	0.010
CDCA4P1	21.039	4.39	0.000
C2CD4D	21.344	4.42	0.021
BBOX1	23.585	4.56	0.039
CSF1R	23.855	4.58	0.020
SGMS1-AS1	24.109	4.59	0.000
FAM71F1	24.169	4.60	0.037
ADRA1D	27.192	4.77	0.005
ERICH3	29.973	4.91	0.016
UBQLNL	35.353	5.14	0.018
CRYM	36.493	5.19	0.008
IL36RN	55.532	5.80	0.022
